# Bridging Biology and Engineering: Unsteady Aerodynamics and Biomimetic Design of Micro Air Vehicles

**DOI:** 10.3390/biomimetics11040250

**Published:** 2026-04-04

**Authors:** Emilia Georgiana Prisăcariu, Oana Dumitrescu

**Affiliations:** Romanian Research and Development Institute for Gas Turbines—COMOTI, 061126 Bucharest, Romania; emilia.prisacariu@comoti.ro

**Keywords:** biomimetic flight, micro air vehicles (MAVs), unsteady aerodynamics, low Reynolds number aerodynamics, flapping-wing propulsion, bio-inspired design, planetary aerial robotics

## Abstract

Micro air vehicles (MAVs) operating at low Reynolds numbers face aerodynamic and structural challenges that differ significantly from those encountered by conventional aircrafts. Nature provides effective solutions to these constraints, as insects, birds, and bats demonstrate highly efficient flight through integrated interactions between morphology, kinematics, and unsteady aerodynamic mechanisms. This review examines how biological flight principles can inform the design of next-generation MAVs. The study first analyzes biological flight strategies across insects, birds, and bats, with emphasis on scaling laws and physiological adaptations relevant to small-scale flight. It then reviews key unsteady aerodynamic phenomena governing low-Reynolds-number flight, including leading-edge vortex stability, wing–wake interactions, tandem-wing effects, and ground influence, as well as current modeling approaches ranging from quasi-steady methods to high-fidelity Navier–Stokes simulations. Building on these principles, the paper discusses biomimetic design strategies for MAV wings, structural–aerodynamic coupling, and actuation technologies used to replicate flapping flight. Existing MAV demonstrators inspired by biological flyers are analyzed, including concepts relevant to planetary exploration environments. Finally, the review identifies current technological limitations and research gaps in materials, actuation, aerodynamic modeling, and system integration. By synthesizing insights from biology and engineering, this work highlights key directions for the development of efficient, adaptable biomimetic MAV platforms capable of operating in complex environments.

## 1. Introduction

### 1.1. Biomimetic Flight and MAV Challenges

Biomimetic design is increasingly important for micro air vehicles (MAVs) because natural flyers (insects, birds, and bats) demonstrate highly efficient and adaptable flight strategies across a wide range of environmental conditions. Insect flight, in particular, shows that flight performance is not defined by aerodynamics alone, but by an integrated system connecting morphology, muscle physiology, energetic capacity, and environmental interactions. Across insect species, the diversity of flight capabilities—such as endurance, maneuverability, load lifting, dispersal capacity, and different life-history strategies—emerges from distinct physiological solutions (e.g., synchronous vs. asynchronous muscles, different metabolic fuels, oxygen delivery pathways, and thermoregulatory capacity) operating within specific aerodynamic regimes [1]. For MAV development, this highlights that optimal performance often requires balancing competing objectives rather than maximizing a single metric.

A key lesson from biology is that flight evolution is shaped by trade-offs and constraints. These may arise from limited resources (e.g., energy allocation between dispersal and reproduction), functional conflicts in biomechanics and control (e.g., stability vs. agility, burst power vs. endurance), and variation in ecological conditions such as temperature, humidity, and atmospheric density [2]. As a result, insects have evolved flight strategies that are highly context-dependent and supported by sophisticated sensing and fast neuromuscular integration. This enables rapid adjustment of wing kinematics in response to wind gusts, turbulence, obstacles, or changing thermal conditions—an approach particularly relevant for MAVs operating at low Reynolds numbers, where disturbances can quickly destabilize the vehicle [3,4,5].

Bird flight provides another major source of biomimetic concepts, particularly for larger MAV scales or hybrid fixed/flapping configurations. Unlike airplanes, birds rely on flapping wings to generate both lift and thrust, achieved through highly adaptable feathered wing structures capable of rapid shape change [6]. Birds can adjust wing camber, area, and orientation in response to immediate flight demands such as cruising, hovering (e.g., hummingbirds), landing, and sudden maneuvering [7,8]. They also use the tail as an active aerodynamic surface to enhance stability, control pitching and yawing moments, and improve maneuverability in complex flow environments [9]. These features motivate the development of morphing wings, flexible structures, and multi-surface control strategies for MAVs operating in cluttered or gusty conditions.

From an aerodynamic perspective, biomimetic inspiration is critical because flapping-wing flight relies on unsteady and three-dimensional mechanisms that differ fundamentally from conventional fixed-wing assumptions. Advances in high-speed videography, computational fluid dynamics (CFD), and particle image velocimetry (PIV) [10,11], together with its later extensions such as stereoscopic and volumetric PIV, have made it possible to quantify complex wing kinematics and directly link these motions to aerodynamic forces and evolving vortex structures in both biological flyers and flapping-wing MAVs [12]. These studies reveal that insects can maintain a stable leading-edge vortex even at high angles of attack, avoiding the stall behavior typical of conventional fixed wings and enabling strong lift production for hovering and aggressive maneuvering [13,14]. Additional force enhancement mechanisms—such as rapid changes in angle of attack at stroke reversal, wing–wake interactions, and interactions between the two wings—further increase aerodynamic efficiency and control authority during flapping cycles [5,13]. Together, these insights support improved analytical and empirical models for predicting instantaneous forces, which are essential for MAV design, control algorithms, and performance estimation.

### 1.2. Ethical Considerations in Bio-Inspired Flight Research

The increasing use of biological inspiration in the design of micro air vehicles (MAVs) raises important ethical considerations related to both experimental methodologies and the broader implications of translating natural systems into engineered applications. As research on flapping-wing aerodynamics often relies on observations of living organisms, it is essential to ensure that experimental approaches minimize disturbance and avoid harm to biological specimens. Advances in non-invasive diagnostic techniques, such as high-speed videography, particle image velocimetry (PIV), and schlieren imaging, have significantly reduced the need for intrusive methods, enabling detailed analysis of flight kinematics and flow structures while preserving natural behavior.

Beyond experimental practice, ethical responsibility also extends to the interpretation and use of biological models [15]. Natural flyers, particularly pollinators such as bees and butterflies, play a critical role in ecosystem stability and biodiversity. Their study should therefore not only support technological innovation but also reinforce awareness of their ecological importance. In this context, bio-inspired engineering can contribute to sustainability by promoting designs that emphasize energy efficiency, adaptability, and minimal environmental impact—principles inherently present in biological systems.

In parallel, increasing attention has been given to the ethical treatment of insects in research, with organizations such as the Royal Entomological Society emphasizing the need to minimize harm, improve transparency, and adopt responsible experimental practices even in the absence of definitive evidence regarding insect sentience [16]. Recent studies indicate that ethical considerations are increasingly influencing field entomology practices, with researchers adapting experimental design and methodologies to reduce unnecessary harm to insect populations [17].

Furthermore, the abstraction of biological mechanisms into engineering frameworks requires careful consideration to avoid oversimplification or misrepresentation of complex living systems. Biological flight emerges from tightly coupled interactions between morphology, physiology, and environmental conditions; isolating individual mechanisms without acknowledging this integration may lead to incomplete or misleading design assumptions. Ethical research practice thus involves maintaining scientific rigor in the translation process, ensuring that biological insights are contextualized appropriately when applied to engineered systems.

Overall, ethical considerations in bio-inspired MAV research encompass responsible experimental methods, respect for ecological systems, and fidelity in the interpretation of biological phenomena. Addressing these aspects strengthens the scientific foundation ofbio-inspiredd design and supports the development of technologies that are not only effective, but also aligned with broader environmental and societal values.

### 1.3. System-Level Behavior and Bio-Inspired Engineering Implications

Biological flight is inherently a system-level phenomenon arising from tightly coupled interactions between morphology, kinematics, structural dynamics, sensing, and the surrounding fluid environment. In contrast to conventional engineering approaches that often isolate aerodynamic, structural, or control aspects, natural flyers operate as integrated systems in which performance emerges from the coordination of multiple subsystems rather than from the optimization of a single parameter. This perspective is particularly relevant for micro air vehicles (MAVs), where low-Reynolds-number conditions amplify the coupling between unsteady aerodynamics, wing deformation, and control dynamics.

At the core of this system-level behavior is the concept of emergence, whereby complex aerodynamic performance—such as stable hovering, rapid maneuvering, and disturbance rejection—results from distributed interactions across spatial and temporal scales. In insect flight, for example, lift generation is not solely determined by wing geometry or flapping frequency, but by the continuous interaction between wing kinematics, vortex dynamics, and body motion. Mechanisms such as leading-edge vortex stabilization, wake capture, and wing–wake interactions do not act independently; instead, they form a dynamically coupled system that adapts in real time to environmental perturbations. This coordinated behavior enables natural flyers to achieve robust performance under highly unsteady and uncertain flow conditions.

Such observations have motivated the development of bio-inspired engineering frameworks that move beyond component-level design toward integrated system architectures. In this context, concepts such as self-organization and distributed control provide useful paradigms for understanding and replicating biological flight. For example, self-organization phenomena have been demonstrated in hardware-oriented models such as two-layer cellular neural networks, where complex global behavior emerges from local interactions between simple units. For example, self-organization phenomena have been demonstrated in hardware-oriented models such as two-layer cellular neural networks, where complex global behavior emerges from local interactions between simple units, enabling the representation of nonlinear and spatially distributed dynamics [18].

Translating these principles to MAV design suggests that effective flight control may emerge from the interaction between passive structural dynamics, local sensing, and adaptive actuation, rather than relying solely on centralized control strategies.

From an engineering perspective, this shift toward system-level design has several implications. First, structural flexibility and passive dynamics should be considered as functional components that contribute to aerodynamic performance and control authority, rather than as disturbances to be minimized. Second, sensing and control strategies may benefit from distributed and event-driven architectures that mimic the fast, localized responses observed in biological systems. Third, the integration of aerodynamic modeling, structural design, and control algorithms should be approached holistically, recognizing that performance metrics such as efficiency, stability, and maneuverability emerge from their interaction.

Ultimately, adopting a system-level perspective enables a more faithful translation of biological principles into engineered systems. By viewing flapping-wing MAVs as coupled aero–structural–control systems, rather than as isolated subsystems, it becomes possible to design vehicles that are more adaptive, resilient, and efficient in complex environments. This paradigm not only bridges the gap between biology and engineering but also provides a foundation for future developments in bio-inspired aerial robotics, particularly for applications involving uncertain or extreme operating conditions.

Despite technological advances, current micro air vehicles (MAVs) still fall short of the performance and adaptability of natural flyers. Limitations include inefficient use of unsteady aerodynamics, restricted maneuverability and lift at low Reynolds numbers, limited control and disturbance rejection, low energy efficiency, and underdeveloped sensing and feedback systems. These gaps underscore the potential of biomimetic design, where insights from insects, birds, and bats—such as unsteady aerodynamics, wing flexibility, morphing structures, and integrated sensory–motor control—can guide the development of MAVs that are more agile, efficient, and resilient in complex environments.

This paper provides a comprehensive overview of bio-inspired MAV design, focusing on aerodynamic principles, wing morphing mechanisms, control strategies, and sensing technologies. We review the state of the art in flapping- and hybrid-wing MAVs, identify persistent design gaps, and discuss how lessons from natural flyers can guide future innovations for agile, energy-efficient, and robust micro air vehicles.

## 2. Biological Flight Principles Across Scales

### 2.1. Insect Flight (Low Re)

**Figure 1:**
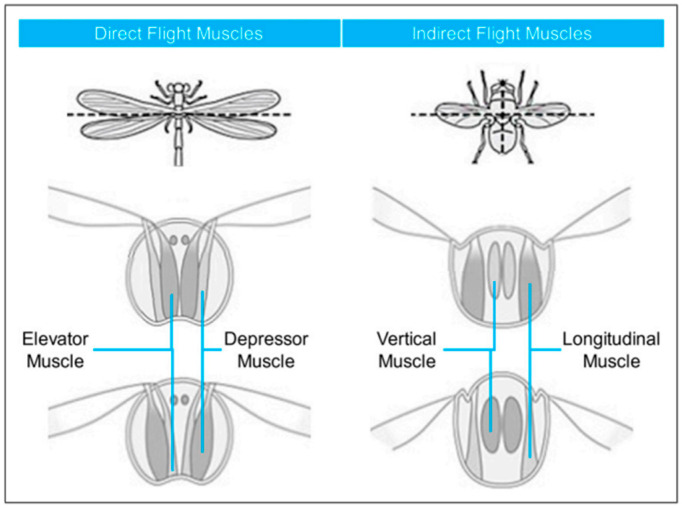
Mechanism of insect muscle during flight [20].

Insects provide key inspiration for understanding flapping-wing aerodynamics due to their remarkable ability to generate lift and maneuver efficiently at low Reynolds numbers. The mechanics of insect flight arise from the interplay between wing kinematics, muscle activity, and aerodynamic phenomena. Insects’ flight muscles are generally divided into direct muscles, which attach directly to the wings and act synchronously with nerve impulses, and indirect muscles, which deform the thorax and act asynchronously [19,20]. These muscle types allow insects to oscillate their wings at optimal frequencies, producing flapping, pitching, and rotational motions necessary for hovering and agile flight; see Figure 1.

Table 1 summarizes the flapping frequencies for different insects, showing that smaller species achieve adequate lift by increasing wingbeat frequency.

Flapping kinematics involve coordinated wing translation and rotation during each stroke, which generates several unsteady aerodynamic mechanisms. During the translational phase, the wings move through the surrounding air while maintaining relatively high angles of attack, whereas during stroke reversal the wings undergo rapid rotation through pronation and supination. These motions generate complex unsteady aerodynamic effects that differ fundamentally from the steady aerodynamics typically observed in conventional fixed-wing aircraft [13].

### 2.2. Bird and Bat Flight (Moderate Re)

Small birds and bats exhibit highly specialized flight adaptations that differ markedly from insects and provide valuable bio-inspiration for MAV design. Both groups have independently evolved powered flight, yet they share common physiological and aerodynamic traits, including low DNA content, fat storage for energy, and habitat-related wing shape adaptations [22,23].

Birds rely on feathered wings that allow rapid adjustments in camber, area, and orientation, while bats use skin-membrane wings capable of substantial flexing during the upstroke and downstroke [24,25]. These morphological differences influence wing morphing strategies: birds primarily adjust individual feathers and wing segments to modulate lift and thrust, whereas bats achieve continuous shape change along the membrane, enabling smoother load distribution and control during flapping. Both strategies support gliding as well as powered flight, allowing energy-efficient transitions between flapping and soaring [26].

Flapping flight remains energetically costly for both groups. Birds and bats of comparable size exhibit similar power requirements, although recent studies suggest that small bats may expend slightly less metabolic energy than previously estimated for small birds [27]. Wingbeat kinematics are tuned to body mass and flight style, with bats generating more complex wake structures and showing adaptations for slow, highly maneuverable flight such as aerial hawking, while birds display broader flight versatility [22]. These differences illustrate how morphological constraints—feathers versus membranes—shape flapping and gliding performance.

To quantify the energetic cost of flapping flight without disturbing behavior, a time-budget method was used to estimate metabolic flight power in small nectar-feeding bats (six species, 7–28 g; 172 daily measurements). Flight power scaled with body mass as metabolic flight power PF = 50.2, mass M 0.771 (r^2^ = 0.96), and was ~20–25% lower than most predictions for small birds, suggesting either overestimated bird flight costs or lower energetic requirements in small bats [27]. A comparison of selected flight-related properties for birds and bats is provided in Table 2.

The combination of wing morphing and precise control enables both birds and bats to achieve remarkable maneuverability and stability. Bats’ flexible membranes allow fine adjustments of wing shape during rapid turns and hovering-like maneuvers, whereas birds exploit feather articulation and tail movements to stabilize flight and respond to gusts or obstacles. Both groups modulate stroke amplitude, wing rotation, and wing camber to balance agility with energy efficiency, demonstrating flight strategies that offer valuable inspiration for MAV designs operating in cluttered or turbulent environments [7,28].

### 2.3. Scaling Laws in Biological Flight

Flapping-wing flight at small scales operates in a fundamentally unsteady aerodynamic regime where conventional quasi-steady assumptions fail to capture the dominant force generation mechanisms. At Reynolds numbers typically ranging from 10^2^ to 10^4^ for insects—and even lower under Martian atmospheric conditions—the flow remains highly viscous, and lift is generated through transient vortex dynamics rather than steady pressure differentials. Central to this regime is the formation and stabilization of the leading-edge vortex (LEV), which remains attached during large angles of attack and prevents classical stall. Additional mechanisms such as clap-and-fling interactions, rotational lift, added-mass effects, and wake capture contribute to force enhancement, particularly during stroke reversal. The relative importance of these phenomena depends on dimensionless parameters including Reynolds number (Re) [29], Rossby number (Ro) [12], and advance ratio (J) [30], which affect vortex formation, LEV stability, and wake–wing interaction dynamics. Tandem-wing configurations, as observed in dragonflies, introduce phase-dependent forewing–hindwing interactions that can improve aerodynamics efficiency and reduce power consumption; these interactions are also sensitive to inter-wing spacing [31,32]. These findings demonstrate that force production in flapping flight emerges from a coupled vortex–structure system, underscoring the necessity of fully unsteady Navier–Stokes-based modeling and high-resolution experimental diagnostics for accurate prediction and engineering translation into micro air vehicles.

At insect scales, lift generation in flapping flight is often described using a first-order quasi-steady scaling of the form

(1) $$F_{z}\sim \frac{1}{2}\rho SC_{L}U_{ref}^{2},$$

where ρ is air density, S is wing area, $C_{L}$ is the cycle-averaged lift coefficient, and $U_{\mathrm{ref}}$ is a characteristic wing velocity. For sinusoidal kinematics, a common approximation is $U_{\mathrm{tip}}∼2\pi fR\Phi$ (characteristic wing tip velocity), where f is the flapping frequency, R wing length, and Φ is the stroke amplitude. This leads to the scaling

(2) $$F_{z}\sim \rho SC_{L}{\left(fR\right)}^{2}$$

illustrating that lift scales quadratically with both flapping frequency and characteristic wing length. However, these relations should be interpreted as scaling approximations rather than constitutive descriptions of natural flapping flight. In insects, aerodynamic force production depends strongly on unsteady mechanisms, including leading-edge vortex attachment, rotational circulation at stroke reversal, wake capture, added-mass effects, and induced vorticity shed into the wake. Accordingly, the total aerodynamic force may be interpreted as the combined effect of quasi-steady and unsteady contributions, rather than being fully represented by a single cycle-averaged lift coefficient and tip velocity. The choice of reference velocity is therefore not unique and may depend on whether the objective is scaling analysis, MAV design, or detailed resolution of instantaneous aerodynamic loads.

However, geometric scaling alone does not guarantee proportional force production, since increasing wingspan typically reduces structural resonant frequency ($f\propto \sqrt{k/m}$) and stroke amplitude under fixed actuation input. Consequently, $U_{\mathrm{tip}}$ and therefore lift may decrease with size unless transmission mechanics or structural stiffness are actively tuned. Recent insect-scale MAV studies confirm that maintaining lift-to-weight ratios $F_{z}/W>1$ across variable wingspans requires compensating for mass growth ($m\propto R^{3}$) by preserving high resonant flapping frequencies or optimizing transmission ratios. These relations demonstrate that flapping flight scaling is governed by coupled aerodynamic, inertial, and structural dynamics rather than simple Reynolds number similarity alone, necessitating integrated aeroelastic and actuation-aware design strategies when translating biological flight principles into engineered micro air vehicles.

The quasi-steady relations used above are consistent with the empirical and modeling framework adopted by Lu et al. [33] for insect-scale flapping MAVs, in which lift production is related to fluid density, wing area, flapping frequency, and stroke amplitude. In that context, the scaling captures the observed tendency for lift to decrease with increasing wingspan unless compensated by transmission-ratio adjustments or high resonant flapping frequencies. Nevertheless, for biological fliers, these quasi-steady relations should be interpreted together with the unsteady aerodynamic mechanisms known to dominate force generation over the wingbeat cycle.

In flapping flight, mechanical power is often approximated by separating an aerodynamic component from the inertial power associated with accelerating and decelerating the wings. For hovering or insect-scale flapping systems, a first-order aerodynamic estimate may be written as [34]

(3) $$P_{\mathrm{aero}}∼F_{z}\;U_{\mathrm{ref}}∼\rho \;S\;C_{L}\;U_{\mathrm{ref}}^{3}$$

where $U_{\mathrm{ref}}$ is often approximated by the tip velocity. This leads to the approximate scaling $P_{\mathrm{aero}}∼\rho \;S\;C_{L}\;(fR{)}^{3}$.

The inertial power arises because the wing has finite mass and moment of inertia I about the flapping axis; if wing angular motion is approximately sinusoidal, the inertial torque scales as $\tau_{\mathrm{in}}∼I\;\ddot{\theta }∼I(2\pi f{)}^{2}\Theta$, and the corresponding peak inertial power scales as

(4) $$P_{\mathrm{in}}∼\tau_{\mathrm{in}}\dot{\theta }∼I(2\pi f{)}^{3}\;\Theta^{2}\propto \;If^{3}$$

showing the same strong cubic dependence on flapping frequency as aerodynamic power.

Under geometric similarity, $I∼m_{w}R^{2}$ (with $m_{w}$ wing mass), so inertial demands can grow rapidly with size unless mitigated by kinematics (lower f), wing retraction, or elastic energy storage. Classic analyses of insect hovering show that without elastic storage, inertial power can dominate the muscle power budget. When wing kinematic energy is partially recovered, the net inertial cost becomes small, leaving mean aerodynamic power as the primary requirement [35].

These expressions are useful as first-order scaling approximations, particularly for hovering insects and flapping MAVs, but they do not represent the only formulation of flight power. Alternative approaches are widely used, especially in vertebrate flight, where total mechanical power is often decomposed into induced, profile, and parasite components following the classical framework introduced by Tucker [36] and later refinements. In such formulations, the relative importance of each term depends on flight speed, morphology, and wake structure. Accordingly, Equations (3) and (4) should be interpreted as simplified scaling relations rather than constitutive expressions applicable to all flapping fliers. Recent insect-scale MAV work reinforces this coupling: to maintain lift and take-off capability across changing wingspans, resonant operation and effective transmission must be preserved so that both $U_{\mathrm{ref}}$ (aerodynamic power) and inertial wing acceleration remain within the actuator’s feasible power envelope [33].

The mechanisms discussed in this section represent physically distinct solutions for flight under different Reynolds-number regimes and kinematic constraints. Insects rely strongly on unsteady lift enhancement, passive pitching, and high-frequency flapping at low Reynolds numbers, whereas birds and bats exploit larger-scale wing morphing, spanwise flexibility, and more complex control of lift, thrust, and maneuverability. From an engineering perspective, these differences provide a functional map for biomimetic MAV development: insect-inspired platforms are particularly relevant for hovering and highly unsteady low-Reynolds-number flight, while bird- and bat-inspired concepts inform morphing wings, compliant structures, and efficiency-oriented designs at larger scales.

## 3. Unsteady Aerodynamics at Low Reynolds Numbers

### 3.1. LEV Stability

At low Reynolds numbers, conventional steady aerodynamic mechanisms become less effective due to early flow separation and reduced lift. Flapping-wing fliers overcome these limitations through unsteady aerodynamic phenomena, among which the stability of the leading-edge vortex (LEV) plays a central role. The formation and sustained attachment of the LEV during the wing stroke enable insects and other biological fliers to maintain high lift coefficients even at large angles of attack [37]. The stability of the LEV is not solely determined by flapping kinematics: in birds, for example, wing morphing and the sweep of the hand wing can generate and stabilize LEVs even during gliding flight [38]. Understanding the mechanisms that stabilize the LEV is therefore essential for explaining insect flight performance and for designing efficient flapping-wing micro air vehicles.

A fundamental concept in unsteady aerodynamics is the delayed growth of circulation, commonly referred to as the Wagner effect [39]. When a wing starts impulsively from rest, circulation increases gradually due to viscous delay at the stagnation point and vorticity shed at the trailing edge, forming a starting vortex. The induced velocity from this vortex temporarily counteracts bound circulation until it moves sufficiently far away, allowing the wing to approach its maximum steady circulation.

**Figure 2:**
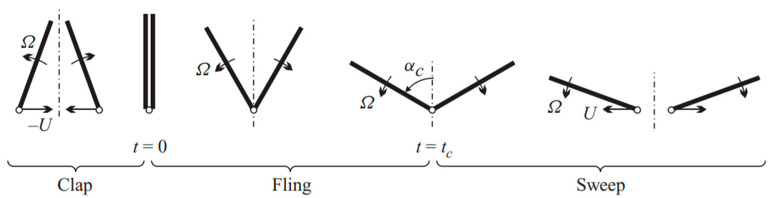
Two-dimensional idealization of the clap–fling–sweep motion of the wings [44].

In flapping flight, several mechanisms interact to produce the high lift coefficients observed in insects, including delayed stall, rotational circulation (Kramer effect), wake capture, and the Weis-Fogh clap-and-fling mechanism [40]. Delayed stall occurs during the translational phase of the wing stroke, allowing the wing to maintain an attached LEV and sustain lift at large angles of attack. Rotational circulation develops when the wing rotates rapidly at stroke reversal, generating additional circulation and contributing to lift production [13,41]. Experimental measurements indicate that delayed stall alone cannot account for the high lift performance observed in insects such as *Drosophila*, highlighting the crucial contribution of rotational mechanisms during wing rotation at stroke reversal [41,42]. Wake capture occurs when vortices shed during one half-stroke interact with the wing during stroke reversal, producing additional aerodynamic forces depending on the timing of wing rotation. Experiments with robotic fruitfly wings demonstrate that variations in rotational timing can significantly influence lift generation [43]. The clap-and-fling mechanism enhances lift and thrust by rapidly building circulation around the wings and partially canceling oppositely signed trailing-edge vortices (Figure 2).

The LEV forms along the wing’s leading edge during each stroke and remains stably attached for much of the motion, preventing stall and enhancing lift [45]. Its formation and stability depend on wing geometry, stroke amplitude, flapping frequency, and rotation timing, and it often interacts with other mechanisms such as wake capture and clap-and-fling [41,46]. Simplified models, including rectangular or cambered plates, are commonly used to study the influence of planform, chord distribution, twist, sweep, aspect ratio, and substructures (e.g., leading/trailing edges, swallowtail structures, and surface textures) on aerodynamic performance and noise generation [5]. Understanding these parameters is critical for translating insect-inspired mechanisms into micro air vehicle design, particularly for achieving stable hovering and high lift-to-weight ratios in low-Reynolds-number regimes.

Wing flexibility further enhances aerodynamic performance. Comparisons between flexible and rigid wings at Re ≈ 10^4^ show that flexible wings stabilize the leading-edge vortex, extend the delayed-stall region, and eliminate transient negative lift observed in rigid wings. As a result, flexible wings generate more vertically oriented aerodynamic forces and improve lift production during hovering [42,47].

Most flying insects possess either a single functional pair of wings or mechanically coupled forewings and hindwings. In contrast, dragonflies have two pairs of wings that can move independently, producing strong aerodynamic interactions between the forewings and hindwings [48]. This tandem-wing configuration enables exceptional flight capabilities, including hovering, speeds up to 54 km h^−1^ [49], rapid 90–180° turning maneuvers [50], and the generation of aerodynamic forces up to 4.3 times body weight [51]. The interaction between the forewings and hindwings is governed by the phase difference, defined as the phase angle by which the hindwing leads the forewing. Adjusting this parameter significantly affects the aerodynamic performance of tandem-wing flapping under different flight conditions [52,53,54].

Key factors influencing LEV formation and stability include wing geometry, stroke amplitude, flapping frequency, and wing rotation. The LEV often interacts with other unsteady mechanisms, such as wake capture and clap-and-fling, which further augment aerodynamic forces [41,46]. These unsteady phenomena allow insects to hover efficiently while maintaining fine maneuverability and stability.

**Figure 3:**
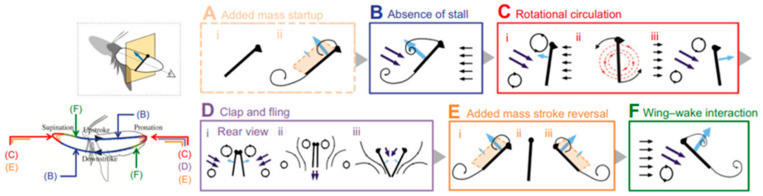
Principal unsteady aerodynamic mechanisms in insect flight: (**A**) added mass effect—transient pressure rise as air accelerates with the wing; (**B**) leading-edge vortex (LEV)—stabilizes flow and delays stall; (**C**) rotational lift—enhanced lift from wing rotation during pronation/supination; (**D**) clap-and-fling—wing interaction at stroke reversal increases circulation and thrust; (**E**) added-mass effects recur during deceleration and re-acceleration; (**F**) wake capture—interaction with the previous stroke’s wake boosts lift. Adapted by Chin et al. [55] from Sane [13].

To compare aerodynamic phenomena across diverse flying animals, Chin et al. [55] introduced three key dimensionless parameters: Reynolds number (Re), Rossby number (Ro), and advance ratio (J). These dimensionless parameters together describe the relative importance of inertial, viscous, rotational, and kinematic effects in flapping flight. Re, calculated from flight speed and a characteristic wing or body length, quantifies the ratio of inertial to viscous forces in the flow; Ro quantifies the effect of wing rotation on the stability of the leading-edge vortex (LEV); and J relates forward flight speed to wing tip velocity, capturing the influence of wing kinematics on propulsion and lift generation. These parameters provide a unified framework for comparing aerodynamic performance across insects, birds, and bats. Figure 3 presents the principal unsteady aerodynamic mechanisms in insert flight.

While leading-edge vortices (LEVs) play a central role in lift enhancement at low Reynolds numbers, their stability and transport mechanisms are strongly Reynolds number-dependent. Experimental studies show that the spanwise flow and vortex transport that stabilize LEVs at Re ≈ 120–1400 differ substantially across Re regimes, and that changes in Re can alter LEV structure and detachment behavior [56]. Consequently, unsteady mechanisms observed in insect flight (Re ≈ 10^2^–10^3^) cannot be directly extrapolated to larger MAVs or bird-scale regimes without accounting for Reynolds number effects on boundary-layer transition, vortex breakdown, and flow coherence [57,58].

Larger Ro indicates stronger spanwise flow, which stabilizes the LEV. The advance ratio (J) quantifies the ratio of forward wing base travel to wing tip travel in the stroke plane during forward flight. J influences wake dynamics, including LEV and tip vortex shedding and reattachment, generally increasing with flight speed. Leading-edge vortex (LEV) stability and lift are strongly influenced by both wing aspect ratio (AR) and Rossby number (Ro). Low-AR wings benefit from strong spanwise flow, which stabilizes LEVs, whereas higher AR reduces this stabilizing effect, promoting earlier vortex detachment and decreased lift [59]. Increasing Ro further destabilizes LEVs regardless of AR, highlighting the importance of considering both parameters together when scaling insect-like unsteady mechanisms to larger MAVs or bird-inspired platforms [60,61]. Time-dependent effects are also critical: transient flow dynamics create an optimal AR range (approximately 3–4) for maximizing lift at flapping amplitudes observed in real insect flight [62]. Direct numerical simulations confirm that lift increases with AR at constant Ro but decreases with increasing Ro at constant AR, representing a balance between competing mechanisms, and similar trends are observed at low Reynolds numbers (Re ≈ 115, fruitfly scale), where viscous effects modify LEV stabilization [63].

**Figure 4:**
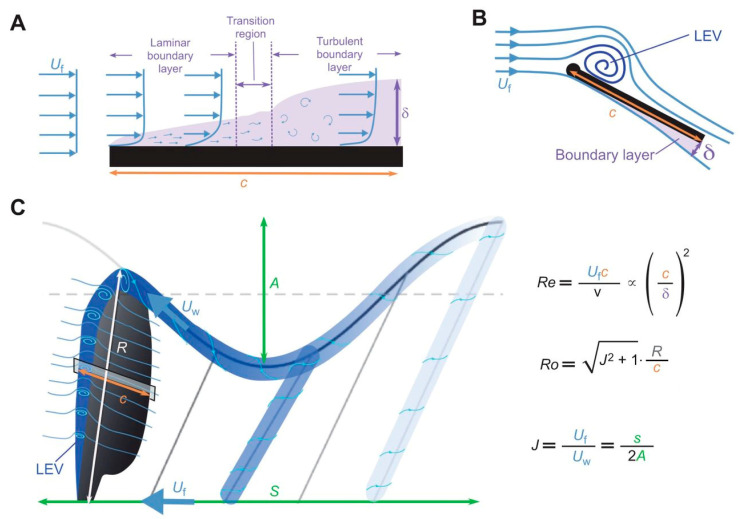
Non-dimensional parameters that determine flapping-wing aerodynamics. Adapted by Chin et al. [55] from Dickson et al. [41].

Together, these parameters offer a comprehensive framework to analyze and compare flapping flight aerodynamics across insects and vertebrates, shedding light on the development of aerodynamic forces and flow structures in varied flight regimes (exemplified in Figure 4).

Table 3 presents a summary of LEV formation mechanisms and effects on lift.

### 3.2. Wing–Wake Interactions

Early research into flapping flight began with studies of insect and bird aerodynamics. In insects, additional lift is generated through wing–wing interactions, most notably the clap-and-fling mechanism. During the clap phase, the wings come together at the end of the upstroke, neutralizing opposing circulations and reducing trailing-edge vorticity. This promotes stronger circulation at the start of the downstroke, enhancing lift. The expelled air during this phase also contributes to thrust, while the motion increases the stroke amplitude, further boosting aerodynamic forces. In the subsequent fling phase, air rushing between the separating wings creates additional lift. These mechanisms recur with each wingbeat, optimized to maximize force production [13,43].

Bird flight, in contrast, is governed by complex unsteady aerodynamic mechanisms arising from flapping wings, which generate wakes filled with vortical structures that encode the time history and magnitude of lift and thrust forces. Traditional theoretical models often idealize these wakes as either closed-loop discrete vortex rings at low speeds or continuous vortex sheets at moderate to high speeds, but high-resolution measurements show that actual wakes frequently exhibit intermediate structures that vary across the wingbeat cycle and flight speeds, containing sufficient momentum to support the bird’s weight throughout a wingbeat [65]. Particle image velocimetry (DPIV) experiments on passerines demonstrate that normalized wake vorticity and circulation decline with increasing flight speed and can be linked to lift production through simple models where normalized circulation represents half the time-averaged lift coefficient [66]. Time-resolved wake measurements across species such as sandpipers, starlings, and robins reveal distinct near-wake vortex patterns during downstroke–upstroke transitions—so-called “double branch” structures—indicating that unsteady shedding and changing circulation gradients actively modulate aerodynamic forces, contributing to lift enhancement and drag reduction [31,67]. Together, these findings highlight that flapping flight performance and efficiency depend on a rich interplay of wake dynamics beyond classical steady-state aerodynamic models.

Lehmann et al. [68] used a dynamically scaled mechanical model of Drosophila melanogaster (known as vinegar fly) to study the clap-and-fling effect on lift. They found that when wing angular separation remains below 10–12°, lift increases by up to 17%, though this benefit depends sensitively on stroke timing. Lift decreases during early clap but rises towards its end [68]. The fling phase exhibits two lift and drag peaks, one dominant at fling onset due to fluid influx, and a smaller one later. Digital particle image velocimetry revealed that leading-edge vortex (LEV) circulation in early fling is lower than inviscid predictions but matches theory later [68]. Wake capture and viscous effects from wing proximity modulate force production, and the clap-and-fling influences wake structure beyond stroke reversal into the upstroke [68,69]. Contrary to earlier views, the clap phase reduces lift transiently, and the overall contribution of clap-and-fling to lift, while modest, likely enhances maneuverability or arises passively from maximizing stroke amplitude, which itself increases forces.

**Figure 5:**
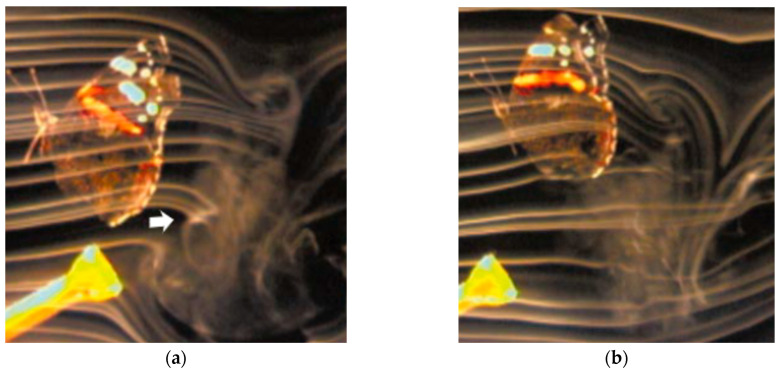
Smoke-wire visualization of wake capture in free-flying *Vanessa atalanta*. (**a**) Downstroke producing a distinct stopping vortex without wake capture, Indicated by the curved streams of smoke at the arrow. (**b**) Subsequent wingbeat interacting with the stopping vortex, disrupting its structure and redistributing momentum in the wake (adapted from [71]).

Free-flight visualizations of *Vanessa atalanta* [70] further clarify the role of wake capture in wing–wake interactions. Using high-resolution smoke-wire techniques in a wind tunnel, Srygley and Thomas [71] demonstrated that at the end of the downstroke, a stopping vortex is shed into the wake. During later wingbeats, the wing can pass through this vortex (wake capture), disrupting it and altering the distribution of vorticity in the near wake. This interaction moves more air while reducing the vortex’s rotational intensity, potentially lowering energetic cost. Flow visualizations show a clear difference: without interaction, a compact, high-velocity stopping vortex remains behind the wing, whereas wake capture creates a larger, more diffuse wake structure (Figure 5). These results show that wing–wake interactions depend strongly on timing and influence both force production and wake structure in flapping flight.

### 3.3. Tandem-Wing Interactions

Insects possessing two functional wing pairs, such as dragonflies and damselflies, exhibit additional aerodynamic complexity arising from forewing–hindwing interactions. Unlike single-wing systems, tandem-wing configurations allow modulation of the phase lag between forewing and hindwing strokes, which directly influences wake structure and force production. Early flow visualizations and dynamically scaled model experiments demonstrated that phase difference strongly alters vortex topology and aerodynamic efficiency [72].

Dragonflies typically employ counter-stroking, with a phase lag of approximately 90°, such that the hindwing begins its downstroke shortly after the forewing [73]. In this configuration, the hindwing interacts with the induced velocity field and shed vortices generated by the forewing. Depending on relative timing and spacing, this interaction may enhance lift through constructive wake coupling or reduce lift due to downwash interference. Willmott et al. showed that vortex loops generated by tandem wings can align vertically, forming a “vortex ladder” structure that increases vertical momentum flux during hovering [73]. Computational studies further confirm that optimal phase lag improves lift-to-power ratios compared with in-phase stroking [74].

The aerodynamic mechanisms governing tandem interactions include modification of effective angle of attack due to forewing downwash, vortex merging between tip vortices of both wings, and transient re-energization of leading-edge vortices on the hindwing. Experiments with robotic and dynamically scaled models reveal that small changes in inter-wing spacing or phasing can shift the interaction from constructive to destructive, significantly altering total force production [72,75]. In-phase stroking tends to produce stronger instantaneous lift peaks but may increase power consumption, whereas counter-stroking often yields improved aerodynamic efficiency. From an engineering standpoint, tandem-wing systems offer a promising strategy for micro air vehicles by enabling phase-controlled force modulation without increasing flapping frequency or stroke amplitude. Adjustable phase lag may provide improved hover stability, redundancy in force generation, and enhanced maneuverability. However, the aerodynamic benefits remain highly sensitive to synchronization and structural alignment, requiring precise control of wing phasing to avoid detrimental wake interference.

**Figure 6:**
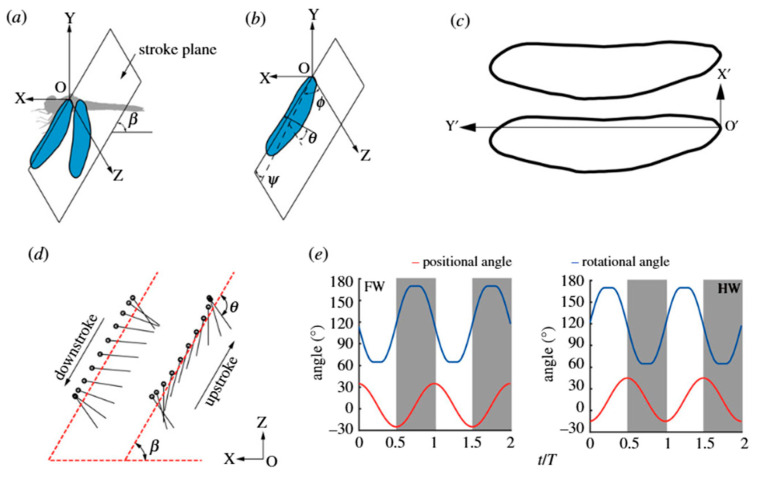
Definition of kinematics and geometry of the FW and HW used in the simulation conducted in [76]. (**a**) Stroke plane and stroke plane angle β for describing the flapping trajectory. (**b**) Three Euler angles of the kinematics of the wing with respect to the stroke plane: the positional angle φ, the rotational angel θ, and the stroke deviation angle ψ. (**c**) The geometry of wings referred to the measurement of Norberg [77]. (**d**) The two-dimensional diagram of wing motion. (**e**) The instantaneous Euler angles for FW and HW for two periods.

To clarify the kinematic framework governing tandem-wing interactions, Peng et al. [76] used Figure 6 to illustrate the geometric and motion parameters used to describe forewing (FW) and hindwing (HW) dynamics in dragonfly-inspired hovering. The stroke plane orientation and stroke plane angle define the reference coordinate system for flapping motion, while the wing kinematics are parameterized through three Euler angles: the positional (flapping) angle φ, the rotational angle θ, and the stroke deviation angle ψ.

The figure further depicts the planform geometry adopted in simulation studies and the temporal variation in positional and rotational angles over two flapping cycles. This representation provides a consistent basis for analyzing phase lag between forewing and hindwing, as well as evaluating how synchronized or counter-stroking motions influence aerodynamic coupling and wake interaction. By explicitly defining these kinematic parameters, the figure establishes the reference framework required to interpret phase-dependent force production in tandem-wing systems.

**Figure 7:**
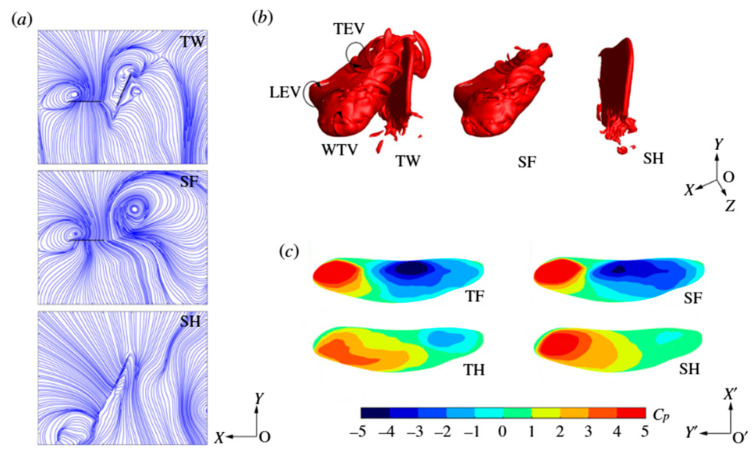
Flow fields and aerodynamic characteristics of tandem-wing hovering at mid-downstroke (T = 0.3) adapted from Peng et al. [76]. (**a**) Streamline distributions at the r_2_ spanwise section illustrating wake interaction between forewing and hindwing. (**b**) Three-dimensional vortex structures showing leading-edge vortices (LEVs), trailing-edge vortices (TEVs), wing tip vortices (WTVs), and tip vortices (TWs), highlighting vortex coupling between tandem wings. (**c**) Pressure coefficient (C_p_) distributions on the upper surfaces of the forewing and hindwing, demonstrating phase-dependent redistribution of aerodynamic loading due to inter-wing interference.

Figure 7 presents representative flow-field and pressure distributions for tandem-wing hovering at mid-downstroke (T = 0.3). Streamline visualizations reveal that the hindwing operates within the induced velocity field generated by the forewing, modifying vortex orientation and wake development. Three-dimensional vortex structures highlight interactions among leading-edge vortices (LEVs), trailing-edge vortices (TEVs), and wing tip vortices (WTVs), while surface pressure distributions (Cp) indicate significant redistribution of suction regions between FW and HW. These results confirm that tandem-wing performance arises from controlled vortex–wake coupling rather than independent force generation.

Phase difference between FW and HW critically affects lift, efficiency, and control. Small offsets (~0–40°) maximize lift for hovering with modest payloads, whereas antiphase motion (180°) produces lift sufficient to support the body weight at lower aerodynamic power, which can extend flight duration relative to other phase settings, without implying unrealistically large payloads [76]. Forewing downwash reduces hindwing lift, whereas hindwing upwash can enhance forewing lift, demonstrating how dragonflies modulate phase to control aerodynamic effects [53]. Experimental work with phase differences of 45–180° shows that hindwing-leading motion minimally affects total lift but significantly influences pitching moments, consistent with observed dragonfly flight behaviors [54].

Wing kinematics and phase coordination strongly influence aerodynamic performance in tandem-wing systems. In-phase stroking tends to produce stronger instantaneous lift peaks but may increase power consumption, whereas counter-stroking improves overall aerodynamic efficiency. Optimal phase coordination is therefore essential for both biological flight and MAV design.

Wing geometry and structural properties also affect aerodynamic performance. Two-dimensional simulations comparing corrugated and flat tandem wings show that longer pitch rotations (80% of the flapping period) produce forces closest to balanced flight, with vertical forces supporting dragonfly weight within 0.92% and negligible horizontal thrust. Corrugated wings differ slightly from flat wings, with vertical and horizontal force variations of ±2.06%, due to differences in vorticity flow patterns [78].

Wing flexibility provides additional aerodynamic advantages in micro-scale MAVs. Experiments with 12 cm-span tandem MAVs indicate that lift increases with angle of attack and is higher for flexible wing skins than rigid skins at low angles (≤10°). At Re = 14,000 and an angle of attack of 50°, flexible wings reached a lift coefficient of 9 [79], highlighting the benefits of flexible-wing designs.

Forewing–hindwing (FW–HW) spacing further influences aerodynamic interactions. Numerical simulations show that increasing hindwing spacing up to 2 c enhances peak horizontal force, whereas spacing beyond 2 c reduces it. Proper adjustment within l_0_ + 2c or h_0_ + 2c optimizes hindwing performance. Additionally, spanwise three-dimensional effects modulate these interactions, producing weaker midspan leading-edge vortices (LEVs) and reducing FW–HW coupling compared with two-dimensional predictions [80,81].

In addition to aerodynamic and structural factors, mechanical and biomimetic tandem-wing mechanisms demonstrate significant lift and stability benefits for MAV applications. Tandem flapping-wing mechanisms with variable beat frequencies (30–210 Hz) and angles of attack (−10–20°) produce approximately 50% more lift than forewing or hindwing pairs alone and improve hovering stability in biomimetic MAVs [82]. Similarly, asymmetric stroke–pitch coupling mechanisms inspired by dragonflies replicate natural-wing kinematics using a single actuator and achieve maximum lift-to-weight ratios of 230.2%, providing useful guidance for the structural and kinematic design of flapping-wing MAVs [83].

Table 4 consolidates key studies on dragonfly-inspired tandem-wing interactions, providing insights into how wing configuration, phase difference, spacing, and kinematics influence aerodynamic performance. Across numerical, experimental, and biomimetic models, several consistent trends emerge:

- Small phase differences (0–40°) or antiphase arrangements enhance lift and efficiency while stabilizing vortical structures;
- Wing corrugation and pitching timing have modest but measurable effects on mean vertical force, indicating that detailed surface features influence lift primarily through local flow control;
- Spacing between fore- and hindwings critically modulates aerodynamic coupling, with optimal spacing reducing detrimental vortex interactions and maximizing total lift. Flexible wings outperform rigid counterparts under moderate angles of attack, confirming the importance of structural compliance for efficient force production.

Overall, the studies collectively highlight that tandem-wing design, phase coordination, and flexibility synergistically govern lift, thrust, and stability, offering clear design guidance for flapping-wing micro air vehicles.

### 3.4. Ground Effect and 3D Flow

This section focuses on computational studies of insect flight, as the lower Reynolds numbers and simpler wing geometries make fully 3D CFD–FSI simulations feasible. In addition, insect-inspired MAVs have been more extensively studied for low-density atmospheres, such as Mars. CFD simulations can provide detailed unsteady flow structures around flapping wings, but due to the complex motion and geometry of bird flight, only limited results have been obtained using RANS and unsteady panel methods, and few high-Re LES studies have been carried out [6].

Studies on ground effect in 3D flapping wings show varied outcomes: hawkmoth wings gain moderate lift-to-drag improvements near the ground, while fruitfly wings show minimal benefit, likely due to Reynolds number (Re) differences (Re ≈ 10,000 vs. 150) [86]. Re varies greatly across species—from ~1 in tiny insects to ~1,000,000 in diving birds—and affects boundary layer behavior, especially in bats and birds at Re > 10,000. However, Re alone does not fully explain unsteady flapping, metrics like Rossby number (Ro) and advance ratio (J) are also used. Comparative wingbeat data reveal that hovering insects (e.g., fruitflies) produce balanced lift and drag on both strokes, while bats and birds generate most force during the downstroke. Hummingbirds, though hovering, still rely more on the downstroke. These differences reflect varying morphology and flight strategies across animal groups.

**Figure 8:**
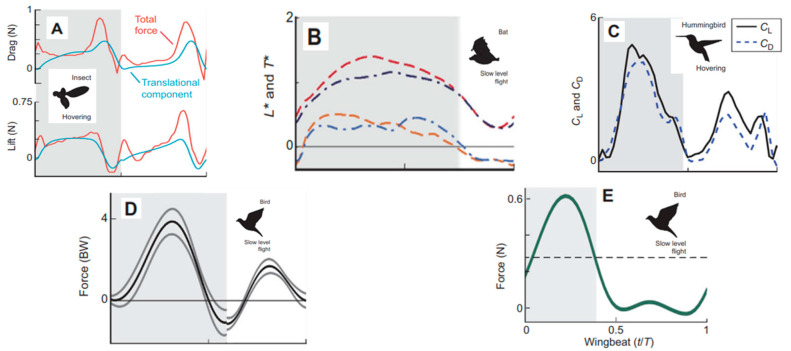
Flapping-wing force within a wingbeat compared among insects, birds and bats: (**A**) Hovering insects produce similar lift and drag in downstroke (gray) and upstroke (white), based on a robotic fruit fly model; (**B**) In *Glossophaga soricina* (dashed) and *Leptonycteris yerbabuenae* (dash-dotted), body-weight-normalized lift (L*) and thrust (T*) mainly occur during downstroke in slow flight; (**C**) Hovering hummingbirds generate reduced but still notable lift and drag during upstroke vs. downstroke; (**D**) Slow-flying pigeons produce much less net aerodynamic force during upstroke than downstroke; (**E**) Pacific parrotlets provide minimal weight support during upstroke in slow forward flight. Adapted by Chin et al. [55] from [87].

Figure 8 illustrates these interspecies force distribution patterns [87]. Dragonflies serve as an excellent model for studying flapping-wing aerodynamics due to their exceptional maneuverability, flight efficiency, and unique ability to independently control their forewings and hindwings. Unlike most insects that flap their wings synchronously, dragonflies can modulate the phase difference (γ) between the forewing and hindwing stroke cycles, enabling a variety of flight modes including hovering, cruising, acceleration, and rapid directional changes. Structurally, dragonfly wings are characterized by low aspect ratio, rigidity, and intricate venation patterns that provide torsional flexibility and structural resilience. These morphological features, combined with distinct kinematic control of each wing pair, allow dragonflies to exploit complex aerodynamic interactions between their fore- and hindwings.

The aerodynamic mechanisms underlying these observations are closely linked to wake–surface interaction and three-dimensional vortex dynamics. In hovering flight, each wingbeat generates a system of interconnected vortex loops composed of leading-edge vortices (LEVs), trailing-edge vortices (TEVs), and tip vortices that close into coherent vortex rings shed into the wake [88]. In free hovering, these vortex rings convect downward, forming a vertically directed momentum jet that supports body weight. However, when operating near a surface, the descending vortex rings interact with the ground, leading to deformation, tilting, and partial reflection of vorticity. Numerical simulations have shown that such interaction can reduce effective induced velocity beneath the wing and temporarily enhance lift when the non-dimensional clearance height (h/R) is on the order of unity [89].

The magnitude and sign of the ground effect benefit depend strongly on Reynolds number, wing kinematics, and stroke timing. Computational studies of hovering hawkmoths demonstrate that near-ground operation can modify pressure distribution and vortex attachment, resulting in moderate lift enhancement under certain kinematic conditions [90]. At lower Reynolds numbers, however, increased viscous diffusion weakens vortex coherence and reduces the impact of ground-induced flow confinement, which may explain the limited benefit observed in smaller insects.

Because these mechanisms are inherently three-dimensional, two-dimensional or quasi-steady models cannot accurately capture the interaction between spanwise flow, vortex stretching, and ground-reflected vorticity. High-resolution volumetric simulations and experimental flow visualizations therefore remain essential for resolving the phase-dependent evolution of wake structures in near-surface hovering [56,91]. For bio-inspired micro air vehicles, clearance height thus becomes a critical design parameter influencing lift generation, stability, and power requirements during take-off, landing, and low-altitude maneuvering.

### 3.5. Modeling Approaches (Quasi-Steady vs. Navier–Stokes)

For over a century, researchers have recognized that quasi-steady flow models cannot fully capture the unsteady aerodynamic forces in insect flight. Simplified flat-plate or blade-element models are commonly used, but direct aerodynamic data from freely flying insects remain scarce. Experiments and smoke visualization studies reveal that insects generate lift primarily through leading-edge vortices (LEVs) and rotational mechanisms at stroke reversal. For example, bumblebees produce lift via LEVs while shedding tip and root vortices independently, unlike species with coupled vortex systems, reflecting a trade-off between maneuverability and efficiency [92].

Insect wings possess complex microstructures, including corrugations, flexible cuticle membranes, and vein networks that act as girder-like supports. These features influence flow separation, vortex formation, stall behavior, and stiffness distribution, affecting aerodynamic performance and deformation during flapping flight [93,94]. Two-dimensional studies show that corrugations can trap vortices, delay stall, and in some cases improve lift and lift-to-drag ratio, depending on flight mode, wing motion, and Reynolds number [95,96]. Three-dimensional analyses of ten insect wings (AR = 2.84–5.45) demonstrate that corrugated and flat wings generate similar aerodynamic forces, as corrugation scales are small relative to LEVs and separated flow regions. While wing shape affects absolute forces, normalized coefficients remain nearly constant, and variations in AR only slightly modify forces due to partial LEV shedding [97].

Experimental and computational studies demonstrate that introducing pitching motion into the flapping cycle can increase lift significantly compared with purely translational strokes, highlighting the critical role of pitch kinematics in vortex generation and control (e.g., rotational circulation interacting with delayed stall and wake capture) that augment lift production beyond quasi-steady predictions [98].

Simplified models, including flat-plate or blade-element approaches, are widely used because they provide fast and computationally efficient predictions of aerodynamic forces [87,88]. These models are useful for the initial design of MAVs but cannot capture LEV formation, rotational mechanisms, wake capture, or wing–root interactions.

- Quasi-steady translation and rotational mechanisms dominate lift production in hovering insects. However, these approximations fail to fully capture lift in species-specific kinematics [13,35,58,59,99]. Power analyses suggest that flight muscle efficiency is limited (~5–9%), and elastic energy storage contributes significantly to hovering energetics [100].
- DPIV and smoke visualization studies reveal vortex structures and demonstrate that wing morphology and flapping motion critically influence lift generation [101].

**Figure 9:**
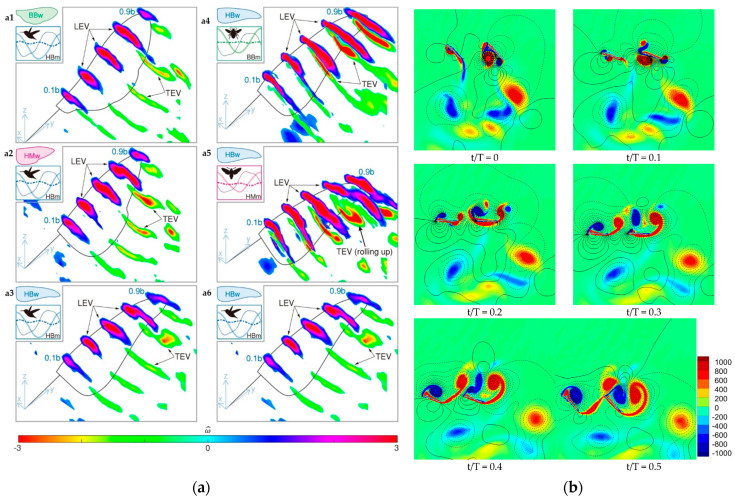
(**a**) Flow-field snapshots showing vorticity and pressure isolines for in-phase stroking wings, (**a1**–**a3**) Comparison of vortex structures of the three different wing shapes at the same flapping motion (hummingbird motion *t⁄T* = 0.26); (**a4**–**a6**) Comparison of vortex structures of the three different flapping motions with the same wing shape (hummingbird wing shape). Bumblebee motion at *t⁄T* = 0.30, hawkmoth motion *t⁄T* = 0.24 of mid-stroke of each flapping motion [101]; (**b**) DPIV results at mid-downstroke, recorded at spanwise sections 0.1b–0.9b for each wing model [102].

Figure 9 displays flow fields of digital particle image velocimetry (DPIV) measurements capturing vortex structures during the mid-downstroke phase for various wing shapes and flapping motions. Analyses show that both wing morphology and flapping kinematics critically influence vortex formation and aerodynamic performance. Vorticity was non-dimensionalized as ω* = ωc/U_tip_, with values ranging from −3 to 3, and measurements were taken at five spanwise sections (0.1b, 0.3b, 0.5b, 0.7b, 0.9b). Comparisons across bumblebee, hawkmoth, and hummingbird wings executing identical or species-specific flapping motions underscore the combined influence of shape and kinematics on aerodynamic forces [101].

Predictive scaling laws and neural network-based dynamic scaling have extended these insights to MAV designs ranging from insect-scale (~1 g) to larger vehicles (~1000 g), validated via full 3D simulations under both terrestrial and Martian conditions [103].

Unsteady aerodynamic simulations employ CFD, fluid–structure interaction (FSI), and hybrid methods to capture wing–flow interactions and vortex dynamics:

- CFD–FSI: Captures detailed interactions including wing flexibility, deformation, and LEV formation [104,105].
- URANS (unsteady Reynolds-averaged Navier–Stokes): Provides time-averaged unsteady flow; may underpredict small-scale vortices.
- LES (large-eddy simulation): Resolves large turbulent eddies, giving accurate vortex dynamics but at higher computational cost.
- DES (detached-eddy simulation): Combines RANS near wing surfaces with LES in separated regions for intermediate accuracy.
- DNS (direct numerical simulation): Resolves all flow scales, but is computationally prohibitive for realistic insect Reynolds numbers.

These high-fidelity approaches allow accurate analysis of LEV formation, wing–wake interactions, and phase-dependent aerodynamic forces, critical for detailed MAV design.

Hybrid approaches balance computational efficiency and accuracy:

- UVLM + optimization algorithms capture general unsteady trends but neglect LEV formation, potentially underestimating lift and power savings [106,107].
- Tandem-wing interference simulations show that wing phase affects lift: in-phase flapping increases hindwing lift by 16%; counter-stroking reduces it by 9% [108].
- Scaling and surrogate models, including ML-based aerodynamic surrogates, extend insights from small-scale MAVs to larger vehicles while preserving key unsteady aerodynamic trends [103].

These approaches are particularly useful for preliminary MAV design, parametric studies, and system-level optimization.

Computational and experimental studies have guided the design of biomimetic MAVs:

- Biplane flapping MAVs use counterphase flapping wings behind a fixed wing to suppress flow separation, achieving stall-resistant flight at 2–5 m/s and a figure of merit of 30 g/W, surpassing low-Re propeller systems by 60% [109].
- Self-stabilizing V-wing MAVs (204 mg, 68 mm wingspan) powered by piezoelectric actuators improved lift by 41.5% and reduced structural asymmetry by 40%, enabling untethered hovering for over 15 s [110].
- Low-Re insect flight (Re ≲ 100) relies on high wingbeat frequency, large stroke amplitude, and partial clap-and-fling for sufficient lift (e.g., leafminer fly, Re ≈ 40) [99].

Table 5 summarizes the main computational approaches for low-Reynolds-number unsteady aerodynamics in insect flight and MAVs. This overview clarifies why fully resolving insect–fluid interactions is challenging and why simplified or hybrid approaches remain widely used.

Building on the biological mechanisms outlined above, the present section examines the aerodynamic principles that make such flight strategies effective at MAV-relevant scales. At low Reynolds numbers, flight performance depends strongly on unsteady flow phenomena that are either absent or less dominant in conventional large-aircraft aerodynamics. Understanding how mechanisms such as leading-edge vortex stabilization, wing–wake interaction, tandem-wing coupling, and ground effect contribute to force generation is essential for translating biological flight strategies into predictive models and practical MAV design rules.

## 4. Biomimetic Wing Design Strategies for MAVs

### 4.1. Planform and Geometry Optimization

Biomimetic wing design for micro air vehicles draws inspiration from natural flyers to address the aerodynamic challenges associated with small-scale, low-Reynolds-number flight. Under these conditions, unsteady mechanisms—particularly the formation, attachment, and stability of the leading-edge vortex (LEV)—dominate force generation. Consequently, geometric parameters such as aspect ratio (AR), camber distribution, and wing planform, together with kinematic features, must be carefully tuned to achieve an optimal balance between lift production and power efficiency during hovering, take-off, and forward flight.

Among geometric parameters, aspect ratio significantly influences LEV behavior and overall aerodynamic performance, although its effect remains strongly coupled with wing planform geometry and flapping kinematics [100]. In an early insect-wing analysis, an optimal AR ≈ 3 was predicted based on the mean lift coefficient. The study showed that as AR increases beyond 3, the LEV grows in cross-sectional size along the span, eventually reaching the trailing edge without reattachment. The interaction with opposite-sign vorticity from the trailing edge weakens the vortex, thereby reducing its lift-enhancing effect [111]. This finding indicates that excessively large AR values can destabilize beneficial vortex structures. Flow-field measurements using robotic flapping wings confirmed this mechanism. Flow measurements with robotic wings show that increasing aspect ratio (AR) strengthens the leading-edge vortex (LEV) but can cause earlier vortex detachment and reduced lift for AR > 5. While circulatory lift rises with AR up to ~6, further increases diminish performance near the wing root and tip, indicating limited aerodynamic benefits beyond this range, consistent with typical insect wings [112].

**Figure 10:**
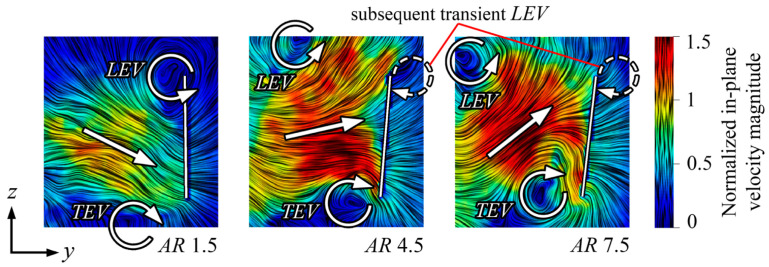
Change in LEV/TEV-induced jet direction (white arrow) with increasing AR; chordwise planes of instantaneous LIC streamlines are illustrated for 75% span at the end of the half-stroke (t* = 0.5, λ* = 6.5); the wing chord is shown by a white line; in plane velocity magnitude is normalized by v_tip_ [112].

Figure 10 illustrates the changes in jet direction (indicated by the white arrow) induced by LEV/TEV as the aspect ratio increases. At lower ARs, the jet is directed downward, whereas, at higher ARs, the jet is tilted upwards in the outboard region of the wing. This change happens because the LEV moves farther from the wing at higher ARs. Additionally, the TEV becomes larger and stronger towards the end of the half-stroke at higher ARs, and when combined with a more pronounced outboard LEV, this generates a stronger induced jet between them [112].

The importance of AR in dictating LEV stability becomes even clearer when extended to higher values. Investigations of revolving hawkmoth planforms (AR = 2.27–7.92) showed that AR had limited influence on overall force coefficients, although higher AR wings exhibited a steeper increase in lift coefficient with angle of attack [113]. However, when AR was increased to 12.5 in simulations of impulsively started rotating wings, the lift coefficient was significantly lower than for AR = 2.5. This reduction was attributed to LEV instability at high AR, where the vortex repeatedly forms and sheds in the outboard region, creating multiple vortex “cells” along the span [114]. Mechanical model studies examining Reynolds number, stroke amplitude, and Rossby number (Ro) have shown that stable LEVs occur when Ro is of order O(1). This implies stable vortex attachment for aspect ratios (ARs) less than 10 [61]. Together, these studies establish AR as a primary parameter governing vortex stability, with an effective operational window roughly between AR ≈ 2 and 6 for flapping-wing MAV applications.

While AR strongly influences LEV behavior, its aerodynamic impact depends on kinematic conditions. A parametric numerical investigation varying AR (1.5–6.0) and r_1_ (0.43–0.63) under insect-scale Reynolds numbers showed that performance trends were largely independent of Reynolds number within the examined range. Lift generally increased with AR; however, aerodynamic efficiency exhibited a clear optimum at AR ≈ 3. Lower AR wings generated insufficient lift, whereas higher AR configurations required disproportionately greater power, reducing power efficiency [115]. The radius r_1_ acted as a secondary tuning parameter: larger r_1_ enhanced lift, but optimal efficiency for a given lift was achieved with combinations of lower r_1_ and higher AR, closely resembling typical insect wing morphologies [93].

The interaction between AR and angle of attack further clarifies these trends. Studies on revolving wings across animal and aircraft AR ranges showed that at high angles of attack (>20°), high AR wings maintain LEV attachment more effectively when the local radius is shorter than four chord lengths. However, at very high AR, the LEV separates in the outboard region. This explains why high AR wings require less power at low angles of attack (as in helicopters), whereas lower AR wings perform better under high-angle hovering conditions (as in hummingbirds) [116]. Thus, optimal AR selection depends not only on geometry but also on operating regime.

In addition to planform parameters, camber distribution significantly affects lift generation and energetic performance. Numerical simulations of chordwise-curved wings in a flapping-wing rotor (FWR) MAV showed that increasing camber height (up to 0.25c) enhances lift. Moreover, positioning the maximum camber closer to the mid-chord region further increases lift, demonstrating the importance of chordwise camber placement [117].

When aeroelastic effects are considered, the role of camber becomes even more pronounced. Passive wing twisting reveals that camber strongly affects thrust and efficiency: a 12.5° camber maximizes thrust, while 15° optimizes propulsive efficiency. Increasing flapping frequency delays twist and requires smaller camber to maintain LEV attachment, but higher thrust comes with greater power consumption, lowering overall efficiency [118]. These results highlight camber angle as a key frequency-dependent tuning parameter that influences vortex structure and overall performance.

The combined influence of AR, camber, taper ratio, and surface area ultimately determines MAV performance. Experimental optimization studies varying camber angle, AR, taper ratio, and wing area demonstrated that both camber and AR significantly affect force production and power efficiency. The best performance was obtained with a trapezoidal wing shape, a straight leading edge, and AR = 9.3, enabling lift-off of a 17.2 g flapping-wing robot. This geometry closely resembled a hummingbird wing, illustrating successful biomimetic translation of biological design principles [119].

Broader UAV-focused studies further examined planform optimization using computational tools such as XFLR5 and Open-VSP in combination with the Analytical Hierarchy Process (AHP). These analyses highlighted the importance of AR, taper ratio, reference area, and stall characteristics. Semi-tapered and moderately tapered wings (λ ≈ 0.5) were identified as optimal for preliminary design. Although highly tapered and elliptical wings generate higher lift, they exhibit less favorable stall behavior, while rectangular wings provide near-elliptical lift distribution but lower lift efficiency [120].

### 4.2. Surface Biomimetics

Biomimetics is central to the design of insect-inspired flying systems, particularly micro air vehicles (MAVs). Scaling biological flight to small vehicles requires integrating a biomimetic design system, which addresses wing morphology, flapping mechanisms, manufacturing, and overall structure, with a control autonomy system to ensure stability and maneuverability [121]. Wing kinematics, including wingbeat frequency, amplitude, stroke plane angle, wing tip path, twist, angle of attack, and camber, provide essential parameters for translating biological motion into aerodynamic performance [41].

A defining feature of insect wings is their surface microstructure, including corrugations, riblets, and flexible profiles. Species such as dragonflies, butterflies, and locusts have rigid veins connected by thin cuticle membranes, forming girder-like structures that influence flow separation, leading-edge vortex (LEV) formation, stall behavior, and stiffness [13]. Two-dimensional studies show that corrugations can trap vortices, delay stall, and in some cases improve lift and lift-to-drag ratio, depending on the flight mode, wing motion, and Reynolds number [47].

Three-dimensional analyses of ten insect wings (AR = 2.84–5.45) demonstrate that corrugated and flat wings generate similar aerodynamic forces, as corrugation scales are small relative to LEVs and separated flow regions. While wing shape affects absolute forces, normalized coefficients remain nearly constant, and variations in AR only slightly modify forces due to partial LEV shedding [122].

Experimental studies using wind tunnels, free-flight arenas, high-speed stereo-photogrammetry, motion capture, and PIV show that natural corrugations have minimal impact on lift at normal angles but help smooth stall at high angles. Wing–wing interactions influence vertical force: counter-stroking reduces hindwing lift due to destructive interference with forewing vortices, whereas in-phase stroking is constructive [123,124]. Tapered wings improve span efficiency by equalizing downwash, and flight performance across cruising, hunting, and territorial modes provides an integrated view of Odonata flight mechanics [82].

Insights from insect biomechanics have informed bio-inspired MAV wing design. Hindwings from three beetle species—Cybister japonicus, Copris ochus, and Harmonia axyridis—were used to develop a pentagonal wing model. Variations in camber, length, chord, AR, taper ratio (TR), and trailing-edge length showed that camber had the largest effect on lift, followed by AR and trailing-edge length. The optimal wing featured 10° camber, 125 mm length, AR = 7.06, TR = 0.40, trailing-edge length 36 mm, and area 4115 mm^2^. Response surface analysis ranked parameters by lift influence: leading-edge length > root chord > trailing-edge length > tip chord [125].

Natural fliers also use passive and active control to resist gusts. Positive wing-beat dihedral stabilizes flight by generating pitch-down and yaw torques, steering the flyer into sideways winds. Smaller dihedral angles in highly maneuverable insects indicate a trade-off between stability and agility. Robotic experiments confirm that adding mass asymmetry to create positive dihedral improves wind stability, offering a bio-inspired approach for robust flapping-wing MAVs [126].

Wind tunnel experiments on whole bio-inspired MAVs show that combining corrugation and flexible profiling improves performance by ~50% compared to flat wings. Low-aspect-ratio wings enhance tip–vortex interactions at high angles of incidence, linking global performance to local flow structures via force measurements and PIV [127].

Most prior studies investigated 2D corrugated wings using physical or computational [128,129,130,131] models, showing that flow separation at ridges forms recirculating eddies that can reduce drag or affect lift. Three-dimensional studies were often limited to chordwise extrusions from a few dried specimens, neglecting spanwise variation, ridge curvature, twist, 3D effects, and interspecies diversity [95]. Real dragonfly wings have distinct corrugations forming a stressed-skin structure of girder-like veins and thin membranes [132].

Corrugated wings improve aerodynamic efficiency in the low-Reynolds-number regime and are particularly relevant for insect-inspired MAVs. Tandem corrugated wings, as found in four-winged insects like dragonflies, benefit from proper fore–hind wing positioning: placing the hindwing below the forewing and minimizing horizontal gaps enhances lift and reduces flow unsteadiness by suppressing wake interactions, achieving efficiencies comparable to a single wing (~10) [132]. Additionally, detailed studies of dragonfly wing corrugations show that positioning corrugations closer to the trailing edge, increasing corrugation amplitude toward the trailing edge, and reducing trailing-edge corrugations all promote coherent leading-edge vortices and reduce lower-surface vortex formation, resulting in lift increases of 20–29% [133,134]. These findings highlight how corrugation geometry and wing arrangement can be optimized to maximize aerodynamic performance for MAV applications.

Table 6, realized by Li et al. [134], highlights gaps in previous research: most studies focused on single factors, such as chordwise position or amplitude, often in 2D airfoils. Few examined gradually varying corrugations or the number of corrugations. This study addresses these gaps by analyzing 3D corrugated wings at dragonfly gliding Re, studying the effects of chordwise position, linearly varying amplitude, and corrugation number.

At Re = 1350, trailing-edge corrugations increased lift by 9.7%, while linearly increasing amplitude toward the trailing edge boosted lift by 28.99% and lift-to-drag by 31.96%. Fewer corrugations consolidated LEVs, improving lift and lift-to-drag by ~20%, showing that strategic corrugation placement, amplitude, and number can optimize low-Re gliding for biomimetic MAV wings [134,135]. At Re = 2150, hovering corrugated wings increased cycle-averaged vertical force by up to 57% compared to flat plates. Tandem corrugated wings strengthen lower-surface vortices and delay stall, enhancing vertical force generation [102].

Studies show mixed effects of corrugation. Hu and Tamai [138] reported better gliding performance compared to flat plates due to suppressed flow separation. Meng et al. [136] found a slight (~5%) reduction in vertical force during hovering due to strong flow separation. Lian et al. [134] observed no lift or stall-delay advantage but noted structural benefits, while Flint et al. [139] reported no propulsion efficiency improvement in pitching wings. More recent studies [83,140,141] indicate that corrugation can increase lift-to-drag ratio by delaying flow separation and stall, particularly in gliding. 

Surface biomimetics, particularly wing corrugations and flexible microstructures, play a critical role in low-Reynolds-number aerodynamics. Properly designed corrugation geometry, amplitude, and number can enhance lift, lift-to-drag ratio, and stall resilience, while tandem-wing arrangements further optimize vertical force and reduce unsteady wake interactions. Although results vary across species and flight modes, these insights provide actionable design guidelines for insect-inspired MAV wings, highlighting the importance of integrating biological form, aerodynamic function, and structural flexibility.

### 4.3. Structural–Aerodynamic Coupling

#### 4.3.1. Bio-Inspired Structural Dynamics

The performance of flapping-wing micro air vehicles is strongly influenced by the interaction between wing structure and aerodynamic forces, a phenomenon widely observed in natural fliers such as insects, birds, and bats. A common bio-inspired approach is passive wing pitching, where the wing root is modeled as a torsional spring, allowing the wing pitch to be determined by the combined action of aerodynamic, inertial, and elastic forces rather than active control [142,143]. This passive mechanism produces wing kinematics resembling insect flight, including characteristic oscillation peaks during each stroke. Proper selection of torsional stiffness is critical: wings that are too rigid or too flexible suffer reduced lift and efficiency, whereas optimally stiff wings can increase average lift by about 10%, enhance instantaneous forces, and produce more concentrated leading-edge vortices [142]. Bio-inspired control strategies, such as adjusting the wing’s rest angle, can generate yaw moments without significantly reducing lift, thereby improving flight maneuverability [144].

The structural design of the wing, particularly the wing root diameter and spar distribution, strongly affects passive pitching behavior. Studies show that flexible wings with a root optimized for passive pitching can achieve a pitch amplitude of approximately 45° and a power economy of 1.26, outperforming rigid wings in efficiency [145]. Similarly, biomimetic wing designs inspired by birds, such as the Sooty Shearwater, demonstrate that careful wing shaping can improve low-speed aerodynamic efficiency. Flow visualization reveals that features such as the division of laminar separation bubbles along the span can enhance lift during stall conditions, highlighting the importance of translating natural-wing morphology into MAV design [146].

Flight control implications of passive pitching are also notable. Compared with active pitching wings, passive wings alter key control derivatives, including vertical force generated by stroke amplitude (ZΦ), flapping frequency (Zf), and pitching moment due to rest angle (Mψ0). For example, increasing flapping frequency does not necessarily increase lift and may even reduce it, emphasizing the need for careful control strategies. Adjusting the rest angle allows modulation of downstroke and upstroke angles of attack, while achieving equal changes requires modifying torsional stiffness [147].

For larger bird-like flapping wings, torsional and bending stiffness play crucial roles in determining lift, thrust, and propulsion efficiency [148]. Optimal aerodynamic performance requires coordination between wing torsional stiffness and the initial geometric twist angle. Spanwise bending flexibility can improve thrust distribution, particularly toward the outer wing, thereby enhancing overall propulsion efficiency [149,150]. These observations have inspired segmented or bifold wing designs in which different wing sections operate with a phase difference to maximize thrust [151].

Aeroelastic modeling and numerical simulations have confirmed these trends. Nonlinear aeroelastic models coupled with fluid–structure interaction methods can accurately predict instantaneous lift, thrust, and spanwise bending and twisting moments, showing good agreement with experimental measurements from flexible flapping wings and ornithopter configurations under both hovering and forward-flight conditions [152,153,154]. Added mass forces associated with wing acceleration are significant, and scaling studies indicate that maximum propulsive force occurs near resonance, whereas optimal propulsive efficiency occurs at roughly half the natural frequency [155]. Membrane wings, which can passively camber and adjust their leading- and trailing-edge angles under aerodynamic loading, further demonstrate that combining structural flexibility with angle-of-attack variation can enhance lift, efficiency, and control during the flapping stroke [156,157]. Excessive camber or non-optimal structural stiffness, however, can reduce aerodynamic performance, highlighting the delicate balance required for optimal flapping-wing design [158].

High-fidelity fluid–structure interaction (FSI) simulations reinforce these conclusions. Flexible bumblebee wings modeled with mass–spring systems coupled to incompressible Navier–Stokes solvers show that wing deformation strongly influences lift and flow dynamics compared with rigid counterparts [159,160]. Similarly, coupled structural–fluid models, employing geometrically exact finite elements and partitioned algorithms, capture the nonlinear dynamics of flexible flapping wings and are validated against experimental hawkmoth wing measurements, confirming the crucial role of flexibility in low-Reynolds-number flight [161]. Simplified methods combining inviscid aerodynamics with linear finite-element models can accurately reproduce lift, thrust, and deformation trends over full flapping cycles, highlighting trade-offs between torsional stiffness, initial geometric twist, and spanwise flexibility [162,163].

Finally, studies of wing compliance and bio-inspired membrane designs demonstrate the practical implications for MAV construction. Vein-like reinforcements in ultra-thin membrane wings allow designers to selectively tailor stiffness and deformation patterns, enabling higher flapping frequencies and modifying force generation in flapping micro air vehicles. Bio-inspired vein patterns change local rigidity and can increase aerodynamic force production, offering pathways to improved thrust and efficiency compared with uniform membrane wings. By strategically distributing stiffness across the wing, it is possible to balance aerodynamic performance with structural requirements, enabling MAV wings that are both lightweight and highly controllable [164,165].

#### 4.3.2. Adaptive Control for Morphing MAVs

While classical control approaches have provided valuable insights into passive and active wing pitching, recent research highlights the potential of neuromorphic and event-driven control strategies to further enhance MAV performance in unsteady and high-frequency aerodynamic environments [166,167]. Neuromorphic control leverages spiking neural networks and bio-inspired architectures to replicate the distributed, low-latency sensory processing observed in insect and bird nervous systems. Such controllers can process local wing deformation and aerodynamic feedback in real time, allowing MAVs to exploit passive structural dynamics rather than compensating for them with purely reactive control [168]. This enables more robust, agile, and energy-efficient flight, particularly under gusty or turbulent conditions where conventional centralized control strategies may be insufficient.

Event-driven control complements neuromorphic strategies by updating actuation only when significant aerodynamic or structural events are detected, rather than at fixed time intervals, enabling ultra-fast, low-latency responses that are vital for high-frequency flapping-wing dynamics in MAVs [169,170]. This approach reduces computational load and energy consumption while allowing faster and more precise responses to sudden changes in aerodynamic forces or wing deformation. For flapping-wing MAVs, event-driven architectures are particularly effective in handling high-frequency aeroelastic oscillations, ensuring that wing morphing and passive compliance are fully leveraged for flight stability and maneuverability.

The integration of these emerging control paradigms with bio-inspired morphing wings, variable-stiffness structures, and 4D-printed adaptive materials presents a promising pathway to optimize the synergy between wing structure and aerodynamics [171,172]. By tightly coupling sensing, actuation, and adaptive control, MAVs can dynamically adjust wing stiffness, torsional pitch, and camber in response to real-time aerodynamic feedback, enhancing lift-to-drag ratios, thrust distribution, and maneuvering capability. Furthermore, such approaches provide a foundation for autonomous operation in challenging environments, from urban wind fields to extreme planetary conditions.

Future research should explore hybrid frameworks that combine neuromorphic, event-driven, and AI-driven adaptive controllers to fully exploit passive and active aeroelastic interactions. Experimental validation on flexible flapping-wing prototypes, including 4D-printed and biomimetic designs, will be crucial to quantify performance gains, energy efficiency improvements, and robustness under realistic flight conditions. These strategies represent a cutting-edge direction for structural–aerodynamic coupling, bridging the gap between laboratory-scale demonstrations and deployable MAV systems capable of advanced autonomy and high aerodynamic efficiency.

## 5. Flapping Mechanisms and Actuation Technologies

Designing bio-inspired flying machines requires replacing the powerful muscles and rapid energy metabolism of animals. Advances in high-energy-density batteries, such as lithium-ion and lithium-polymer, have made electric actuators the primary power source for most micro air vehicles (MAVs). Common actuators include brushed and brushless DC motors, typically paired with gearboxes and linkage or cable mechanisms to produce flapping motions. Alternative approaches include direct-drive systems exploiting resonance and efficient piezoelectric actuators operating at flapping frequencies. Some designs mimic the resonant insect thorax by using electromagnetic actuators to achieve large flapping amplitudes with low energy consumption.

Over time, various configurations and control strategies for flapping-wing drones have been developed to improve maneuverability, stability, and efficiency. Researchers have explored multiple approaches to mimic the complex wing motions observed in nature, including independent wing actuation, wing twist modulation, and amplitude-offset control methods. Among these, Karasek [173] developed a hummingbird-sized, tailless flapping-wing MAV capable of hovering using independent wing motion for flight control. Two control methods were tested: wing twist modulation inspired by the Nano Hummingbird, and a novel approach based on flapping amplitude and offset modulation via linkage joint displacement. Both produced roll, pitch, and yaw moments exceeding simulation targets. The twist-based method offered simplicity and low actuation forces but required specific wing designs. The amplitude-offset method was compact and wing-independent but suffered from high reaction forces and joint instability.

Limitations of bulky micro servos and underperforming shape memory alloys underscored the need for improved actuators for stable, efficient control. Sivasankaran et al. [174] developed an electromagnetic flapping-wing actuator powered by a 9 V DC supply. A stable oscillation signal was generated with an LM555 oscillator circuit, with frequency and duty cycle controlled by resistors and a capacitor. This signal drove a Power MOSFET switch, which actuated a miniature relay for wing motion. Adjustable wingbeat frequencies reached up to 250 Hz. Wings were glued to a flat iron plate connected to the actuator, mimicking a dragonfly’s joint structure.

**Figure 11:**
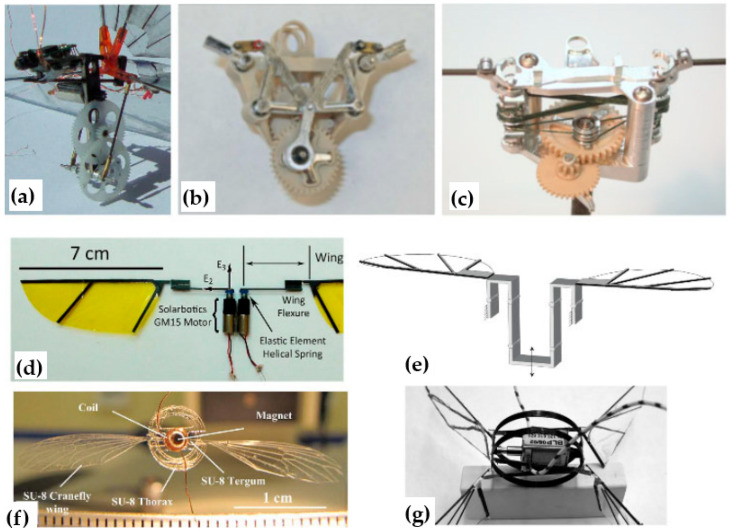
Examples of flapping mechanisms [173]: (**a**) DelFly Micro mechanism [175], (**b**,**c**) Nano Hummingbird and cable mechanism [176], (**d**) direct drive mechanism [177], (**e**) compliant mechanism of Harvard robotic fly [178], (**f**) flexible resonant wing [179] and (**g**) resonant thorax [180].

The mechanism produced a linear up–down stroke with a 60° flapping angle, replicating dragonfly hovering. Liang [82] introduced a flapping-wing mechanism replicating dragonfly stroke and pitch motions. Using a single DC motor with a custom transmission system coupling stroking and pitching, this design addressed the limitations of conventional mechanisms controlling only one degree of freedom. The transmission includes driving and driven gears, a constrained transmission shaft, and linked joints driving the wing (Figure 11). Experimental validation confirmed accurate asymmetric wing motion. Aerodynamic tests showed a maximum lift-to-weight ratio of 230.2%, highlighting its potential for efficient bio-inspired aerial vehicles.

Another study [81] used an electromagnetic actuator powered by 12 V DC to drive biomimetic micro air vehicle (BMAV) wings. A stable oscillation was generated via an LM555 circuit, with frequency controlled by resistors and a potentiometer, enabling wingbeat frequencies from 30 to 210 Hz. BMAV wings were 3D-printed using materials such as HIPS and Ultrat, which demonstrated nanomechanical properties close to real dragonfly wings. HIPS was preferred for its superior surface finish and consistent melting during printing on a MakerBot. Wings were attached to small iron plates glued to the actuator for repeatable flapping motion.

**Figure 12:**
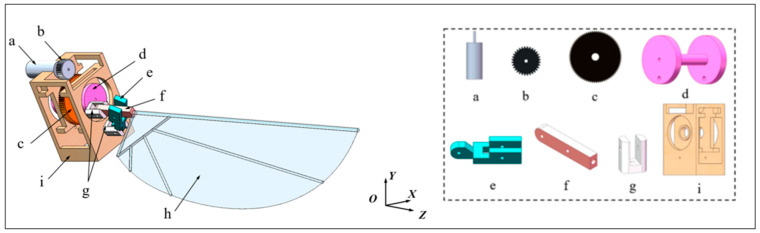
Exploded view of the flapping-wing mechanism: a—motor, b—driving gear, c—driven gear, d—transmission shaft, e—linkage, f—linkage, g—rotational axis, h—flapping wing, i—frame [82].

Flapping-wing robots typically use separate actuators for wing flapping and tail/wing control. Flapping is most often driven by brushless DC motors converting rotation via reduction gears and crank or cam mechanisms into reciprocal wing motion (Figure 12). Servomotors are less common for flapping due to limited response speeds, causing reduced motion range at high frequencies [181].

For tail or rudder control, servomotors are preferred for their simplicity and torque (1.5–4.3 kg/cm) [182,183], usually under 15 g. Continuous actuation using smart materials is an emerging bio-inspired approach allowing dynamic wing tip adjustments to modulate lift and improve maneuverability [184,185,186]. Onboard sensors support both localization and mission data collection.

**Figure 13:**
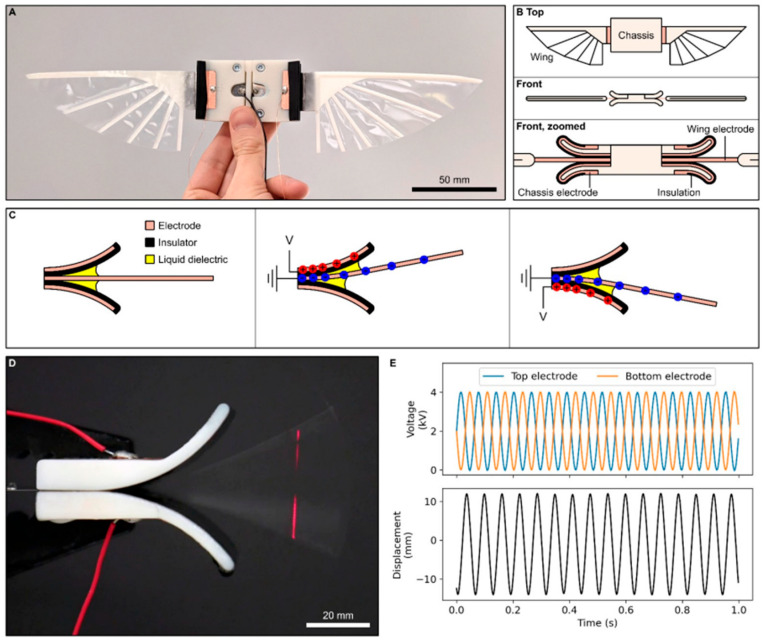
Liquid-amplified zipping actuators for micro air vehicles with transmission-free flapping: (**A**) example of a transmission-free flapping MAV prototype; (**B**) transmission-free flapping MAV design; (**C**) Actuation principle of the Liquid-amplified Zipping Actuator; (**D**) LAZA during testing, showing wing electrode oscillation at 23 Hz; (**E**) Time-varying voltage applied to the top and bottom electrode, and resultant movement of the central electrode. Maximum voltage *Vmax* = 4 kV [184].

An innovative example of compact and transmission-free actuation is the Liquid-Amplified Zipping Actuator (LAZA) developed at the University of Bristol (Figure 13). This system employs electrostatic attraction between flexible electrodes separated by a high-permittivity liquid dielectric, converting electrical energy directly into mechanical flapping motion without gears or linkages. The liquid medium amplifies displacement and force, enabling high specific power and long fatigue life within a lightweight, sealed structure. Such actuators demonstrate how soft, liquid-enhanced electrostatic mechanisms can deliver efficient, controllable motion at small scales offering a promising concept for adaptive or bio-inspired systems in aerospace and robotic applications [184].

**Figure 14:**
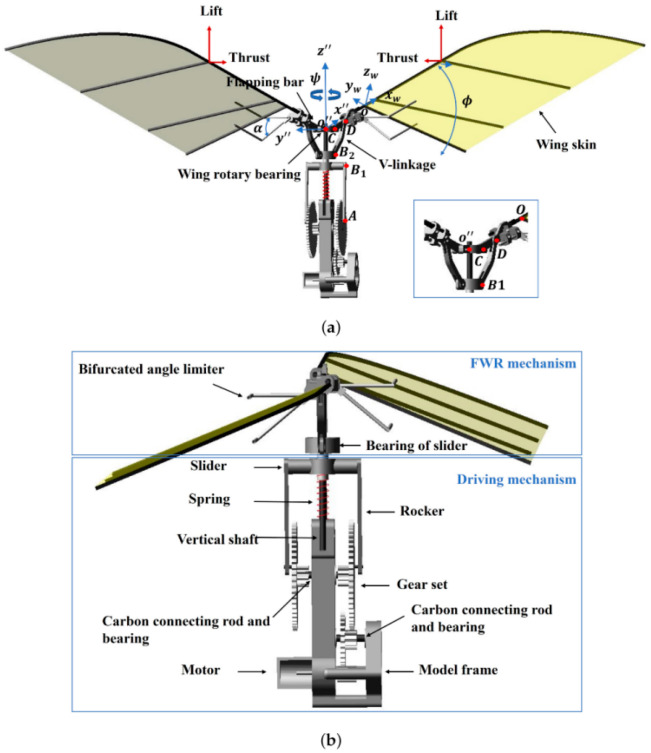
The FWR model of flapping and rotating motions about a shaft with a spring: (**a**) front view and (**b**) side view [185].

The FWR is powered by a 3.7 V DC motor with a 19.5:1 double-reduction gearbox, driving a pair of 330 mm span wings mounted symmetrically on a vertical shaft through a V-shaped linkage (Figure 14) [185]. The wings undergo active flapping and passive pitching, constrained by an angle limiter and governed by aerodynamic and inertial forces. Lift and thrust are generated via inverse Kármán vortices, while opposing thrusts induce rotation about the central shaft. The FWR motion is described by three rigid-body angles: rotation, flapping and pitching.

While numerous bio-inspired flapping-wing concepts have been proposed in the past decade, none has achieved a fully integrated structural–thermal–optical validation chain that simultaneously characterizes mechanical deformation, convective heat dispersion, and flow-field evolution. Most published results rely on simplified geometries or purely numerical studies, resulting in limited physical correlation and low technology maturity (TRL < 3). Furthermore, existing prototypes are seldom optimized for manufacturability in controlled or space-analog environments, where materials, actuation mechanisms, and optical diagnostics must operate under thermal, vacuum, or microgravity constraints. These gaps motivate the present activity, which aims to establish a reproducible experimental and numerical framework bridging these domains.

4D printing, an extension of additive manufacturing, introduces the dimension of time, enabling structures to adapt their shape or properties in response to external or internal stimuli such as heat, light, electricity, magnetism, solvents, or pH [186]. This capability relies on smart materials—including shape memory polymers, alloys, hydrogels, ceramics, piezoelectric elements, and electroactive polymers—that allow controlled deformation, variable stiffness, and multifunctional behavior [187]. In the context of biomimetic wings and flapping-wing micro air vehicles (MAVs), 4D printing provides a unique opportunity to integrate structural compliance, actuation, and morphing capability within lightweight architectures, enabling improved aeroelastic adaptability, efficiency, and maneuverability [188,189]. Drawing inspiration from bio-inspired morphing—such as the adaptive wing shapes of birds, compliant membranes of bats, and fin stiffness modulation of fish—research has shown that shape-changing structures can enhance aerodynamic performance, reduce drag, and increase lift-to-drag ratios by up to 30%, while also improving propulsive efficiency and reducing noise [7]. The technology offers additional benefits familiar from conventional additive manufacturing, including rapid prototyping, design flexibility, weight reduction, and compatibility with multifunctional composites [190]. However, the field remains at an early stage: key challenges include fabrication precision, fatigue resistance, energy efficiency, multi-stimuli integration, and the development of robust, field-ready systems [191,192]. Despite these limitations, the combination of 4D printing and smart materials represents a transformative route toward adaptive, multifunctional, and structurally integrated biomimetic wings, bridging the gap between laboratory prototypes and future high-performance autonomous MAVs in aerospace, robotics, and soft-robotic applications.

## 6. Biomimetic MAV Demonstrators

### 6.1. Earth-Based Insect-Scale MAVs

**Figure 15:**
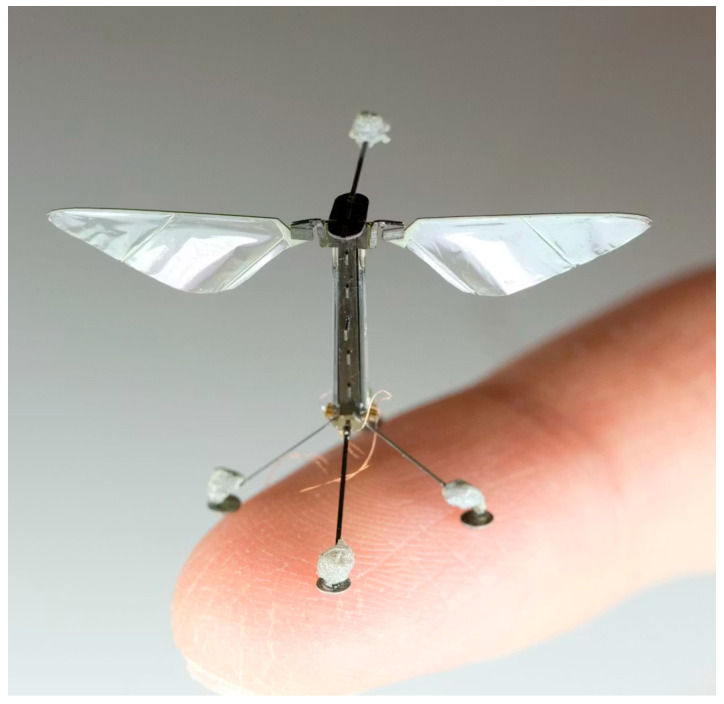
RoboBee: Insect-inspired robots with potential uses in crop pollination, search and rescue missions, surveillance, as well as high-resolution weather, climate, and environmental monitoring [193].

The RoboBee Project [193], developed at the Harvard Microrobotics Laboratory [194] within the Harvard John A. Paulson School of Engineering and Applied Sciences [195], is a pioneering research initiative focused on the development of insect-scale flying robots inspired by the biomechanics of bees. Weighing less than a gram and powered by piezoelectric actuators, RoboBee achieves controlled flapping-wing flight at high frequencies, operating in low-Reynolds-number regimes similar to biological insects. The platform explores distributed sensing, autonomous control, perching, water landing, and swarm coordination, with applications ranging from environmental monitoring and search-and-rescue to precision agriculture. Beyond terrestrial use, RoboBee (Figure 15) serves as a testbed for studying bio-inspired aerodynamics, artificial muscle actuation, and microfabrication techniques—contributing foundational knowledge to the fields of microrobotics, morphing structures, and adaptive flight systems.

**Figure 16:**
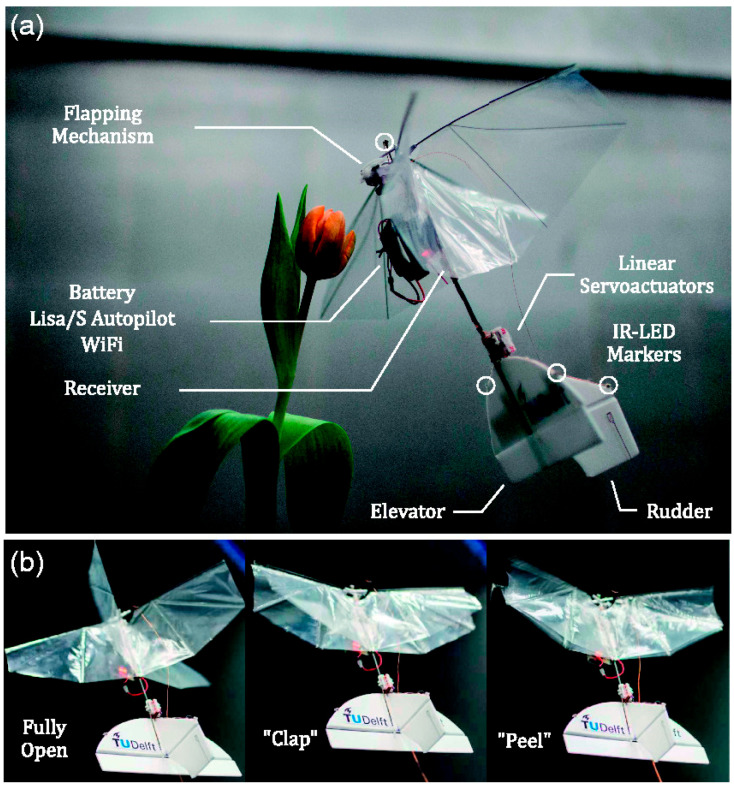
The DelFly II FWMAV used in the tests. (**a**) Description of the main components. (**b**) The important phases of the flapping motion, including the “clap” and “peel” which help enhance the lift production and efficiency. For reliable tracking, the DelFly was equipped with four active IR-LED markers, three placed on the tail and one on the nose [198].

Another representative prototype has been during the DelFly Project [196], developed at Delft University of Technology, which is a leading European initiative in flapping-wing micro air vehicles (MAVs). Inspired by dragonflies, DelFly platforms such as DelFly II (Figure 16) [197], DelFly Explorer [198], and DelFly Nimble [199] combine lightweight flexible wings with stereo vision and onboard autonomy to achieve stable hovering, agile maneuvering, and fully untethered flight. Operating at low Reynolds numbers, DelFly vehicles exploit clap-and-fling aerodynamics and unsteady vortex dynamics to generate lift efficiently at small scales. The project has demonstrated autonomous obstacle avoidance and indoor navigation, making it a benchmark platform for bio-inspired propulsion, vision-based control, and morphing-wing aerodynamics in micro-scale flight systems.

### 6.2. Mars-Relevant Concepts

This section does not aim to provide descriptive project context, but to synthesize existing biomimetic MAV concepts and translate them into a system-level design perspective relevant for future aerial robotics, including extraterrestrial environments. By comparing conceptual and implemented platforms, this section identifies recurring design patterns, limitations, and unaddressed challenges, thereby bridging the gap between biological inspiration, aerodynamic theory, and system-level MAV implementation. Conceptual flight scenarios and mission illustrations are not intended as speculative visuals, but as engineering abstractions used to contextualize aerodynamic and system-level constraints under non-terrestrial conditions (e.g., low-density atmospheres). Such representations are commonly used to guide early-stage design and to evaluate feasibility against known physical scaling laws.

Marsbees are a NASA NIAC (NASA Innovative Advanced Concepts) concept developed in the 2010s for flapping-wing, insect-inspired flyers capable of operating in the ultra-thin Martian atmosphere. The concept was led by a collaborative US–Japan team, combining expertise in microrobotics, numerical modeling, and optimization for low-density atmospheres. The system envisions a Mars rover as a mobile base that deploys a swarm of “Marsbees,” small bumblebee-sized robotic flyers with cicada-sized wings, equipped with sensors and wireless communication. The rover functions as a recharging and communication hub, while the Marsbees enable reconfigurable sensor networks, resilient systems, and collaborative sample or data collection. Key innovations include insect-like compliant wings, high lift achieved through dynamic similarity, and a torsional spring mechanism to reduce power consumption. The Marsbees concept demonstrates flexibility, robustness, and accessible testing, representing a versatile approach to Martian aerial exploration [199,200].

**Figure 17:**
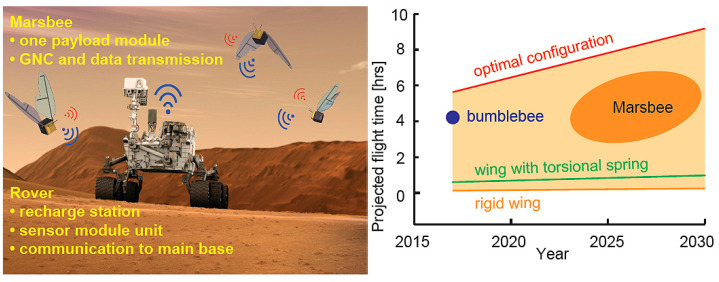
Marsebee design and project flight timeline [199].

Figure 17 presents Marsbee concept and the project flight timeline.

**Figure 18:**
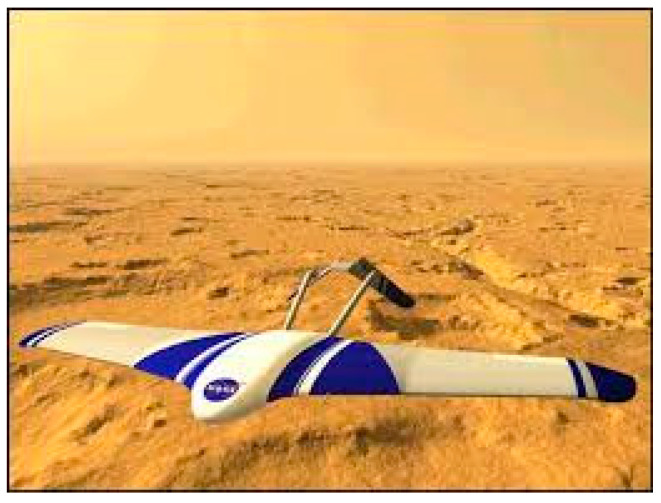
Artist’s conception of ARES performing its survey over the surface of Mars [202].

The Aerial Regional-scale Environmental Survey (ARES) was a NASA Langley Research Center proposal in 2005 for a robotic-powered airplane capable of sustained flight over the Martian surface to collect atmospheric and surface data. Selected as a finalist for the Mars Scout program, ARES envisioned an aircraft flying several kilometers above the surface to perform scientific observation, including atmospheric profiling and remote sensing. The proposal highlighted the potential for regional-scale environmental surveys on Mars that could complement surface-based rovers [201]. A concept of ARES is presented in Figure 18.

**Figure 19:**
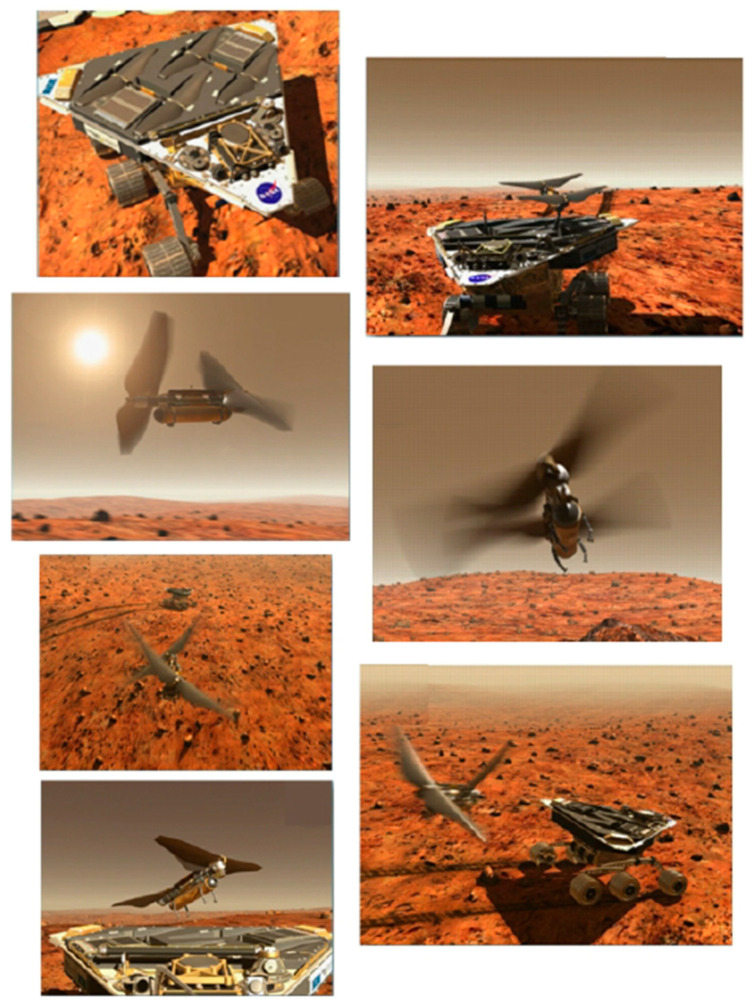
Entomopter flight mission [204].

Entomopter is a flapping-wing aircraft developed at the Georgia Institute of Technology starting in 1996. The project received initial internal research and development (IRAD) funding, with subsequent support from DARPA, the Air Force Research Laboratory (AFRL), and the NASA Institute for Advanced Concepts. The Entomopter employs insect-inspired wing-flapping aerodynamics and a “blow-wing” mechanism for lift enhancement. Variants include the solar-powered Solid-State Aircraft, demonstrating that flapping-wing vehicles can achieve sustained flight with lightweight, low-power systems. The research showed promise for small-scale autonomous aerial exploration on Mars or other planetary surfaces [203]. This mission sequence is shown in Figure 19.

The European Space Agency (ESA) has also promoted the development of flapping-wing propulsion technology for future Mars surface explorers. Their vision includes lightweight, insect-like systems ranging from bumblebee to hummingbird scales, capable of autonomous operation and in situ measurements, pushing the boundaries of micro aerial vehicle (MAV) technology for extraterrestrial applications [205].

**Figure 20:**
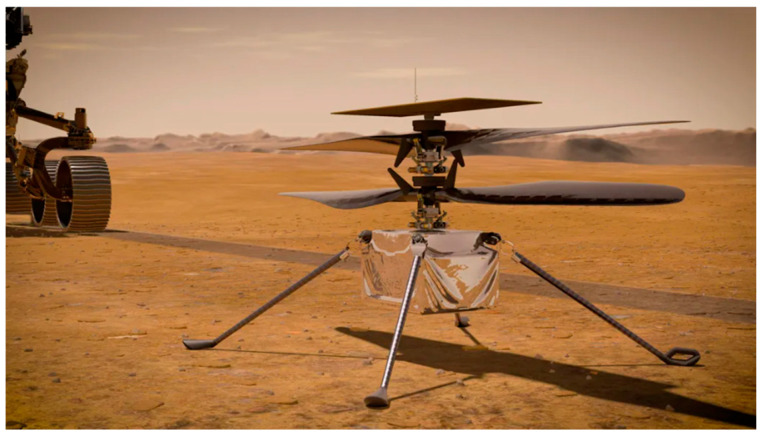
NASA’s Ingenuity Mars helicopter [207].

While not biologically inspired, NASA’s Ingenuity helicopter has already demonstrated the feasibility of powered flight in the Martian atmosphere (Figure 20). First flown in April 2021, Ingenuity serves as a technology pathfinder for future MAVs or lightweight aerial systems, validating key flight control, power, and sensing technologies in the ultra-thin Martian environment [206].

Table 7 summarizes key concepts, vehicles, and achievements in planetary and Mars-focused aerial exploration, highlighting the evolution from early insect-inspired flapping-wing prototypes to small helicopters and future flapping-wing MAV designs. It includes the developers, vehicle scale, and notable technological accomplishments, illustrating how bio-inspired flight and micro air vehicle concepts have been adapted for extreme planetary environments.

## 7. Current Challenges and Research Gaps

Translating biological insights into deployable MAVs requires addressing not only laboratory-scale validation but also practical constraints such as energy density, robustness, and multi-agent coordination. For extreme or planetary applications, scaling laws, environmental interaction, and adaptive control must be explicitly considered. This emphasizes the importance of unifying biological principles, aerodynamic mechanisms, and engineering design within a coherent, physically grounded framework.

### 7.1. Limitations in Biomimetic MAVs

Due to their small size, low weight, and operation at low Reynolds numbers, MAVs experience complex aerodynamic phenomena that differ significantly from those of conventional aircrafts. Despite significant progress in design methodologies, materials, propulsion systems, and control strategies, several technical limitations remain fragmented, with advances in individual domains outpacing system-level integration. Aerodynamic performance optimization at low Reynolds numbers, efficient energy storage and propulsion integration, flow control strategies, and robustness under gusty conditions continue to represent major research challenges. Moreover, the scaling effects associated with miniaturization introduce interdisciplinary constraints involving aerodynamics, structural dynamics, and control systems.

A central structural weakness in biomimetic MAV research is the imbalance between modeling sophistication and experimental validation. High-fidelity CFD and reduced-order unsteady aerodynamic models are increasingly common, yet robust, time-resolved experimental datasets at MAV scales remain limited. The issue is not merely scarcity of data, but lack of standardized benchmark geometries and repeatable measurement protocols. Force measurements at sub-gram scales, synchronized deformation tracking, and high-speed PIV are technically demanding and rarely reproduced consistently across laboratories. As a result, validation is often case-specific rather than generalizable. Models may appear predictive within isolated studies, but their external robustness remains uncertain. This limits cumulative knowledge building and slows convergence toward validated aerodynamic frameworks.

A related structural limitation is the dominance of low-TRL prototypes. Many flapping-wing MAV platforms demonstrate proof-of-concept flight but remain highly tuned laboratory systems. Optimization frequently targets feasibility—achieving hovering or controlled motion under constrained conditions—rather than operational robustness. Environmental disturbance rejection, long-duration stability, and manufacturability are often secondary considerations. Consequently, each new prototype advances capability incrementally but does not necessarily raise the technological baseline of the field. The transition from experimental demonstrator to deployable platform remains structurally underdeveloped.

Control architecture presents another systemic gap. Biological flight systems rely on tightly integrated sensing–actuation loops with minimal latency, exploiting direct coupling between structural deformation and neural feedback. Research inspired by Mandyam Srinivasan’s work [208] on how insects use optic flow to regulate flight and guide navigation, and discussed in broader MAV contexts by Dario Floreano [209] in the context of insect-inspired autonomous aerial systems, highlights the efficiency of optical flow-based regulation in insects. In contrast, most MAVs rely on centralized, PID-based control structures in which sensing and actuation are sequentially separated. This segmentation introduces latency and bandwidth limitations, particularly problematic in flapping-wing systems where aerodynamic forces vary at high frequencies. The absence of embedded, neuromimetic feedback prevents MAVs from exploiting aeroelastic responses as part of the control loop. As a result, control remains reactive rather than reflexive, reducing robustness and increasing energy expenditure.

Energy constraints amplify these structural weaknesses. Endurance bottlenecks are not isolated battery problems; they shape the entire system architecture. The limited energy density of lithium-based storage forces strict mass budgets, restricts sensing redundancy, and limits computational overhead. High-frequency actuation—required for insect-scale flapping—further increases power demand. Flight durations are often too short to evaluate long-term stability or degradation, restricting the ability to perform statistically meaningful performance assessments. Endurance thus becomes a systemic constraint that influences aerodynamic design, control strategy, and experimental methodology simultaneously.

Mass–power scaling further restricts miniaturization. Aerodynamic force production scales with surface area, while structural mass, embedded electronics, and power systems do not decrease proportionally. At smaller scales, actuator efficiency declines due to electromagnetic and frictional losses. The result is a nonlinear scaling imbalance: reducing size does not proportionally reduce energy demand. As emphasized in analyses synthesized by Wei Shyy, aerodynamic feasibility alone does not guarantee energetic viability. This scaling constraint imposes fundamental limits that incremental optimization cannot fully resolve.

Structural bio-inspiration, particularly wing corrugation, illustrates a recurring disconnect between biological observation and engineering realization. Corrugated wings enhance the stiffness-to-weight ratio and influence low-Re flow behavior, yet artificial reproduction often captures geometry without replicating anisotropic mechanical properties. Microfabrication approaches, including those developed in insect-scale robotics by Robert J. Wood, achieve high geometric precision but remain costly, multi-step, and difficult to scale. The aerodynamic benefits of corrugation depend on compliant structural response; rigid approximations therefore under-realize biological functionality.

Repeatability compounds these issues. Flapping-wing MAVs are highly sensitive to micro-scale variations in stiffness distribution, hinge compliance, mass balance, and actuator phase alignment. Minor deviations can shift resonance behavior and aeroelastic phase lag, significantly altering flight performance. Without consistent manufacturing pipelines, validation becomes prototype-specific, and comparative studies lose statistical significance. Repeatability is therefore not simply a production concern but a methodological limitation that restricts theoretical consolidation.

### 7.2. Future Directions

Table 8 synthesizes the primary technological and methodological barriers limiting progress in biomimetic micro air vehicles (MAVs), identifies the underlying structural causes of each limitation and clarifies their broader system-level consequences. The table emphasizes how deficiencies in validation, scaling, fabrication, and control integration interact to constrain performance, reproducibility, and scalability beyond laboratory-scale demonstrations.

Current research is addressing several key challenges through innovative approaches, such as reinforcement learning [210] for real-time aerodynamic control in turbulent conditions, biomimetic design inspired by bird flight [211] to optimize wing efficiency and stability, and CFD-based rotor [212] configuration optimization to enhance lift force production and maneuverability. Additionally, data-driven models and surrogate-based optimization techniques [213,214,215] are being used to refine airfoil designs and improve overall aerodynamic performance. Together, these developments suggest that the field is gradually shifting from isolated proof-of-concept studies toward more integrated and adaptive design methodologies.

The system-level constraints summarized in Table 6 should be viewed not only as limitations but also as interconnected research opportunities. Priorities include the following:

- Standardized experimental protocols and open, time-resolved datasets to strengthen validation, improve reproducibility, and enable reliable cross-study comparisons;
- High-fidelity numerical frameworks capable of resolving unsteady aerodynamics, fluid–structure interaction, and aeroelastic coupling at MAV scales with improved computational efficiency;
- Machine learning and AI-based approaches, including surrogate and reduced-order modeling, real-time state estimation, reinforcement learning, adaptive estimation, and physics-informed learning, for distributed control and effective exploitation of aeroelastic interactions through integrated sensing–actuation loops rather than centralized architectures.

At the technological level, transition-oriented design strategies are essential to move beyond low-TRL demonstrators toward platforms with cumulative technological maturity. Endurance and miniaturization bottlenecks call for advances in lightweight power systems, efficient actuators, scaling-aware design, and energy-aware control strategies. Manufacturing innovations, including microfabrication, material-compatible corrugation design, and repeatable micro-assembly, remain critical to realize bio-inspired geometries with sufficient fidelity and reproducibility.

The combination of these research directions provides a clear roadmap from current prototypes to ambitious applications. For example, 4D printing and smart materials can enable morphing wings with embedded compliance and actuation [189]; bio-inspired morphing and variable-stiffness structures improve aerodynamic efficiency, lift-to-drag ratio, and maneuverability [7,216]; and AI-driven adaptive control ensures robust, energy-efficient flight in dynamic, unpredictable environments [217,218]. Together, these approaches directly address the technical bottlenecks highlighted in Table 6 and provide feasible pathways toward deployable MAVs capable of planetary exploration, long-duration autonomous missions, and operation under extreme conditions.

The integration of standardized experiments, high-fidelity simulations, adaptive bio-inspired structures, smart material-enabled morphing, and intelligent control frameworks transforms the current limitations into opportunities, paving the way for robust, reproducible, and application-ready biomimetic MAVs that can meet both near-term research goals and visionary long-term missions.

## 8. Conclusions

Biological flight continues to inspire the development of micro air vehicles operating at low Reynolds numbers. This review demonstrates that integrating unsteady aerodynamic mechanisms, optimized wing morphology, and compliant structural designs allows natural flyers to achieve levels of efficiency, maneuverability, and adaptability that remain challenging to replicate with conventional engineering. Advances in experimental techniques, high-fidelity computational methods, and novel materials are progressively enabling researchers to better understand these mechanisms. Current numerical approaches, including CFD coupled with fluid–structure interaction, large-eddy and direct numerical simulations, and hybrid blade-element or surrogate models, allow detailed prediction of wing–flow interactions and vortex dynamics. Meanwhile, developments in lightweight, high-frequency actuators, flexible wing structures, and advanced sensing systems are enabling MAVs to fly independently.

In addition, recent trends in artificial intelligence and machine learning are enabling data-driven aerodynamic modeling, predictive control strategies, and rapid optimization of wing kinematics and MAV designs. Surrogate models trained on high-fidelity simulations or experimental data allow efficient exploration of the design space, guiding actuator selection, phase coordination, and material placement. At the same time, advanced fabrication techniques such as 4D printing offer dynamic, adaptive wing structures that can change shape or stiffness in response to aerodynamic or environmental loads, mimicking biological morphing and further enhancing flight performance.

Despite significant progress, key challenges remain. These include further improving actuator efficiency and power density, integrating robust sensing and control in extremely small platforms, and achieving predictive yet computationally efficient aerodynamic modeling. Continued interdisciplinary collaboration across biology, aerodynamics, materials science, and robotics will be essential to overcome these limitations. Biomimetic MAVs are expected to play an increasingly important role in aerial robotics, with applications ranging from environmental monitoring and search-and-rescue to surveillance and infrastructure inspection.

Looking further ahead, bio-inspired aerial platforms may also become critical for planetary exploration. Small flapping-wing vehicles, inspired by insects and birds, could offer unique advantages for navigating complex terrains, caves, or atmospheric boundary layers inaccessible to traditional rovers or larger aircrafts. By combining the efficiency and adaptability observed in nature with advances in aerospace engineering (numerical modeling, AI-driven optimization, adaptive materials, and miniaturized actuators), biomimetic MAVs are poised to open new pathways for scientific exploration both on Earth and beyond.

## Figures and Tables

**Table 1:** Insect flapping frequencies [21].

Insect	Wing Size [mm]	Frequency [Hz]
Butterflies	42.7–57.3	4–10
Damselfly	18–190	15–20
Dragonfly	50–127	25–40
Beetles	14–25	40–90
Honeybee	9.7	200
Mosquito	2.4–3.3	450–600
Midges	1–3	600–1000

**Table 2:** Comparison of some selected properties between birds and bats. “=” indicates that birds and bats show similar values and “≠” indicates that they differ in this property [22].

Property	Birds	=/≠	Bats
Body mass (kg)	0.002–15	≠	0.002–1.5
Aspect ratio	3–15	≈	5–14
Wing loading (N m^−2^)	4–160	≠	4–60
Span ratio	0.2–1	≠	~0.7
Wing tip path	anticlockwise	≠	clockwise
High lift (CL > 1.6)	yes	=	yes
Power curve	U-shape	=	U-shape
Power scaling (W)	56 mass 0.75	=	55.7 mass 0.80
Flight speed (m s^−1^)	15.9 mass 0.13	≠	8.4 mass 0.08

**Table 3:** Leading-edge vortex (LEV) formation mechanisms in flapping flight.

Mechanism	Description	Effect on Lift	Key Factors
Delayed Stall [13,41,42]	Wing maintains attached LEV during translational stroke	Sustains lift at large angles of attack	Wing geometry, stroke amplitude, Re
Rotational Circulation (Kramer Effect) [13,41]	Rapid wing rotation at stroke reversal generates extra circulation	Adds lift at stroke reversal	Wing rotation timing, flapping frequency
Wake Capture [53]	Vortices shed in previous half-stroke interact with wing	Produces additional aerodynamic forces	Timing of stroke reversal, wing position
Clap-and-Fling (Weis-Fogh) [40]	Wings clap together and fling apart at stroke reversal	Enhances lift and thrust, partially cancels trailing-edge vortices	Wing tip separation, stroke amplitude
Wing Flexibility [42,47,64]	Deformation under aerodynamic/inertial loads	Stabilizes LEV, extends delayed stall, reduces negative lift	Wing material, Re ≈ 10^4^, planform
Tandem-Wing Interaction [48,49,50,51,52,53,54]	Forewing and hindwing interact with phase difference	Modulates lift and maneuverability	Phase angle γ, wing spacing
Spanwise Flow (Ro) [55]	Rotational flow along span stabilizes LEV	Maintains vortex attachment, increases lift	Rossby number, wing rotation
Forward Flight (J) [55]	Forward motion relative to wing tip velocity affects wake	Influences LEV and tip vortex shedding and reattachment	Advance ratio J, flight speed

**Table 4:** Summary of key studies on dragonfly-inspired tandem-wing interactions.

	Wing Configuration	Phase/Timing	Key Results
Tandem-wing hovering, γ = 0–180° [76]	Tandem	Phase 0–180°	Small phase (0–40°) → lift > 20% body weight, high efficiency; γ = 180° → supports body weight with low power; stable vortex structure enhances efficiency.
Phase & pitching moments [54]	Tandem	Hindwing leads 45–180°	Total lift/thrust mostly unchanged, but pitching moment varies significantly; matches dragonfly antiphase and forward-flight patterns.
2D tandem-wing sections [78]	Tandem, corrugated vs. flat	Wing pitch rotation 80%, 60%, 40%	80% rotation produces forces closest to balanced flight; mean vertical force supports 0.754 g within 0.92%; horizontal force negligible; corrugated wings ±2.06% differences from flat wings.
Tandem MAV, 12 cm wingspan [79]	Flexible vs. rigid	Flapping 11 Hz	Lift increases with AoA; flexible wings outperform rigid wings at AoA ≤ 10°; max CL = 9 at 50° and Re = 14,000.
FW–HW spacing effect [80]	Tandem	γ = N/A	Peak horizontal force on hindwing increases up to 2c spacing, then decreases; optimal spacing l_0_ + 2c or h_0_ + 2c improves performance.
3D effects on LEV [81]	Tandem	0–180°	Spanwise LEV weaker at midspan than 2D; reduces vortex interaction, lowering fore–hindwing aerodynamic coupling.
Tandem flapping-wing mechanism [82]	Tandem	Variable (30–210 Hz)	Tandem wings produce roughly 50% more lift than fore- or hindwing alone; enhanced stability for hovering.
Asymmetric stroke–pitch mechanism [83]	Tandem	Stroke–pitch coupled	Accurately replicates dragonfly kinematics; max lift-to-weight ratio 230.2%; large aerodynamic benefit for MAV design.
Corrugated doubly tandem wings [84]	Tandem, corrugated	N/A	Delays or suppresses trailing-edge separation; thick tandem wings achieve 1376% higher efficiency at 0° AoA; hindwing decalage increases lift.
Tandem flapping wings [85]	Tandem	Hindwing position & polar angle varied	Lift and thrust strongly depend on hindwing arrangement; optimal positioning can increase lift by ~78% with minimal thrust loss. Wing–wake interactions reduce negative upstroke forces, enhancing average lift. 2D simulations; 3D effects not included.

**Table 5:** Computational tools for unsteady low-Reynolds-number flapping flight.

Computational Tool	Accuracy	Limitations	Modeling Technique	FSI Coupling	Example Applications	Relative Computational Cost	Usage in the Field	Key Flow Features Captured	Validation
RANS/URANS	Captures mean unsteady forces and large-scale flow structures	Underpredicts small-scale vortices; cannot fully resolve wake dynamics	Reynolds-averaged Navier–Stokes with turbulence models (e.g., k–ω, k–ε)	Can be coupled with FSI but often simplified	Hovering insects, preliminary MAV design	Low	Very high—most common for preliminary studies	Large-scale vortices, average lift and drag trends	Medium—validated for mean forces, not detailed vortices
LES (Large-Eddy Simulation)	Resolves large unsteady vortices; good for vortex dynamics	High mesh and time resolution required; limited to small domains or short time	Resolves large energy-carrying eddies; subgrid-scale model for small turbulence	Can be coupled with FSI for deformable wings	Insect flapping wings, wake analysis	High	Moderate—used for detailed vortex/wake studies	LEVs, tip vortices, wake interactions, vortex shedding	High—validated in low-Re insect studies
DES (Detached Eddy Simulation)	Hybrid: RANS near walls, LES in separated regions; balances cost and fidelity	Still expensive; less accurate near complex surfaces than full LES	Combines RANS (near-wall) + LES (separated region)	Feasible; can capture unsteady loads with FSI	MAV wings with partial separation, hovering insects	Medium–High	Low–moderate—some studies on MAVs with partial separation	LEVs, wake interactions, separated flow regions	Medium—limited experimental validation
DNS (Direct Numerical Simulation)	Full resolution of all flow scales; highest fidelity	Computationally prohibitive for realistic MAV or insect wings; limited to very low Re	Full Navier–Stokes without turbulence model	Fully compatible; best for fundamental studies	Fundamental vortex studies, LES/URANS validation	Very High	Rare—mainly for fundamental studies at very low Re	All flow scales including small vortices and wake dynamics	Very high—used for fundamental benchmark studies
Reduced-Order/Low-Order Models	Fast, captures trends in lift, drag, and wake	Cannot resolve detailed vortex structures; limited to specific operating conditions	Simplified physics-based (lifting-line, blade-element) or data-driven ROM	Can include simplified FSI effects	MAV design, optimization studies, parametric sweeps	Very Low	High—widely used for MAV design and optimization	Approximate LEV effects, average wake forces	Medium—validated against experiments or CFD data

**Table 6:** Comparison between this study and prior research on corrugated wings [134].

		Dimensionality	Corrugation	*Re*
Chordwise corrugation position	Xu [135]	2D	At leading, middle, and trailing edge	1500
	Meng [136]	3D	Close to the leading edge	1000
	Rohit [137]	2D	At leading and trailing edge	1000
	Li [134]	3D	At leading and trailing edge	1350
Corrugation amplitude	Zhang [138]	2D	Global variation in all corrugation amplitudes	500~12,000
	Shabbir [139]	2D	Amplitude variation in the first two leading-edge corrugations	58,000~125,000
	Wang [140]	3D	Global variation in corrugation amplitude	50,000~100,000
	Li [134]	3D	Chordwise linear variation in corrugation amplitude	1350
Number of corrugations	Rohit [137]	2D	Variation in the number of leading-edge corrugations	1000
	Li [134]	3D	Variation in the number of trailing-edge corrugations	1350

**Table 7:** Overview of notable insect-inspired and small-scale aerial vehicles for planetary exploration.

Year/Period	Concept/Vehicle	Developer/Organization	Type/Scale	Key Results/Achievements
1996 onward	Entomopter	Georgia Institute of Technology, funded by DARPA, AFRL, NASA NIAC	Insect-inspired flapping-wing aircraft	Developed “blow-wing” lift enhancement; solar-powered Solid-State Aircraft variant; demonstrated feasibility of small, low-power flapping-wing flight for planetary exploration
2005	ARES (Aerial Regional-scale Environmental Survey	NASA Langley Research Center	Robotic powered airplane	Selected as Mars Scout finalist; designed for sustained flight several km above Martian surface; enabled atmospheric profiling and regional surface observation
2010s	Marsbees	NASA NIAC (US–Japan collaboration)	Bumblebee-sized flapping-wing swarm	Combined Mars rover base with swarm of small flyers; featured insect-like compliant wings, high lift through dynamic similarity, torsional spring for power reduction; enabled reconfigurable sensor networks, collaborative sampling, and resilient exploration
2021	Ingenuity helicopter	NASA JPL	Small helicopter (~1.8 kg)	First powered flight on Mars; validated flight, navigation, and sensing in ultra-thin Martian atmosphere; serves as technology pathfinder for MAVs and lightweight aerial systems
Future/ESA call	ESA flapping-wing MAVs	European Space Agency	Insect- to hummingbird-scale	Development of lightweight, autonomous flyers for in situ measurements and exploration on Mars; focus on flapping-wing propulsion and planetary MAV operations

**Table 8:** System-level constraints and structural gaps in biomimetic MAV development.

Core Issue	Structural Cause	System-Level Consequence
Lack of high-fidelity validation	Non-standardized experiments, scarce time-resolved datasets	Weak external validity of models
Low-TRL prototypes	Optimization for demonstration rather than deployment	Limited technological accumulation
Limited neuromimetic feedback	Segmented sensing and actuation	High latency, reduced robustness
Lack of integrated loops	Centralized control architectures	Inefficient exploitation of aeroelastic coupling
Endurance bottlenecks	Low energy density, high-frequency actuation cost	Constrained autonomy and testing duration
Mass–power scaling	Nonlinear scaling laws, actuator inefficiency	Fundamental miniaturization limits
Corrugation fabrication limits	Geometry–material mismatch	Under-realized bio-inspired gains
Repeatability	Manual micro-assembly, high sensitivity to tolerances	Poor reproducibility and validation uncertainty

## Data Availability

All generated data are contained in the article or can be made available upon request.
